# Extensive Variation in Gene Copy Number at the Killer Immunoglobulin-Like Receptor Locus in Humans

**DOI:** 10.1371/journal.pone.0067619

**Published:** 2013-06-28

**Authors:** Sanne Vendelbosch, Martin de Boer, Remko A. T. W. Gouw, Cynthia K. Y. Ho, Judy Geissler, Wendy T. N. Swelsen, Michael J. Moorhouse, Neubury M. Lardy, Dirk Roos, Timo K. van den Berg, Taco W. Kuijpers

**Affiliations:** 1 Sanquin Research and Landsteiner Laboratory, Department of Blood Cell Research, Amsterdam, The Netherlands; 2 Sanquin Blood Supply Foundation, Department of Immunogenetics, Amsterdam, The Netherlands; 3 Emma Children’s Hospital, Department of Pediatric Hematology, Immunology and Infectious Diseases, Academic Medical Center, Amsterdam, The Netherlands; Kyushu Institute of Technology, Japan

## Abstract

Killer immunoglobulin-like receptors (KIRs) are involved in the regulation of natural killer cell cytotoxicity. Within the human genome seventeen KIR genes are present, which all contain a large number of allelic variants. The high level of homology among KIR genes has hampered KIR genotyping in larger cohorts, and determination of gene copy number variation (CNV) has been difficult. We have designed a multiplex ligation-dependent probe amplification (MLPA) technique for genotyping and CNV determination in one single assay and validated the results by next-generation sequencing and with a KIR gene-specific short tandem repeat assay. In this way, we demonstrate in a cohort of 120 individuals a high level of CNV for all KIR genes except for the framework genes *KIR3DL3* and *KIR3DL2*. Application of our MLPA assay in segregation analyses of families from the Centre d’Etude du Polymorphisme Humaine, previously KIR-genotyped by classical techniques, confirmed an earlier reported duplication and resulted in the identification of a novel duplication event in one of these families. In summary, our KIR MLPA assay allows rapid and accurate KIR genotyping and CNV detection, thus rendering improved transplantation programs and oncology treatment feasible, and enables more detailed studies on the role of KIRs in human (auto)immunity and infectious disease.

## Introduction

The immune system is a complex network that protects the human body against internal and external threats. It consists of an innate and an adaptive system. Natural killer (NK) cells constitute the first line of innate immunity against viral infections and tumor development. Recent studies have shown that NK cells can also be educated during development and that memory NK cells are able to mount a more effective cytokine response upon reactivation, suggesting adaptive functions [1], [2].

The importance of human NK cells has become clear through studies concerning cancer, viral infections and donor/recipient panels in transplantation settings. The cytotoxicity of NK cells is regulated through large families of molecules with a homologous structure, belonging to the C-type lectin-like molecules and immunoglobulin-like receptors [3].

The most prominent family that regulates NK cell cytotoxicity in humans comprises the killer immunoglobulin-like receptors (KIRs). Binding of a KIR to one of its ligands, Human Leukocyte Antigens (HLA) class-I molecules and possibly others, triggers either an activating or an inhibiting signal, thereby controlling the immune reactivity of NK cells.

A widely studied application is the matching of KIRs in donors and recipients in transplantation settings [4]–[7]. Alloreactive NK cells can lead to rejection of organs after transplantation [7]. In contrast, donor KIR-ligand mismatching with the recipient during hematopoietic stem cell transplantation (HSCT) may be required for the beneficial effects of NK cell alloreactivity against tumor cells [5], [8]. Apart from inhibitory KIR-ligand mismatches, the presence of activating KIR genes in the donor grafts has been shown to improve survival after unrelated HLA-matched HSCT [9], [10]. These studies and others have shown that a favorable combination of HLA and KIR genotyping of both donor and recipient can strongly enhance the chance of success of transplantation.

The genes for the KIR family are present on human chromosome 19q13.4 [11]. In humans, seventeen highly homologous genes, including two pseudogenes, have been identified to date. For all of these genes a large number of allelic variants exists, which resemble each other up to 98% in sequence homology. Distribution of the genes is thought to occur on two basic haplotypes, designated A and B, both of which contain the same so-called framework genes (*KIR3DL3*, *KIR3DP1*, *KIR2DL4* and *KIR3DL2*). Other KIR genes may or may not be present on one of the haplotypes [12], [13]. In addition, individuals may carry more than two copies of a KIR gene, as has been shown so far only in case of *KIR3DL1* and *KIR3DS1* [14]–[16]. The immunological relevance of copy number variation (CNV) in the *KIR3DL1* and *KIR3DS1* genes was shown by Pelak and co-workers ^15^, who described that a higher number of genes relates to a better individual resistance against human immunodeficiency virus type 1 (HIV-1). This resistance was related to a higher number of KIR3DS1 protein-expressing NK cells and an inhibition of replication of HIV-1 [15]. The importance of CNV in relation to disease was also supported by the correlation of increased clearance of hepatitis C virus (HCV) in individuals with of two copies of *KIR2DL3* compared to those with one or no copies [17].

Tools to study the extent and functional meaning of this inter-individual KIR locus variation on a larger scale are currently lacking. From other immune receptors we know that extensive CNV exists and that the number of genes can relate to protein levels, leading to a difference in disease outcome. We have observed this for the genes encoding the human Fc-gamma receptors (FcγR) and complement factor 4 [18]–[20]. To study the level of CNV in the KIR gene cluster, we have used a convenient, sensitive and efficient MLPA-based method to detect all KIR genes in one assay. The introduction of a synthetically derived calibrator has allowed us to accurately quantify KIR gene CNV. Validation of the method by comparing the MLPA method with the standard polymerase chain reaction (PCR) with sequence-specific primers (SSP) in a large cohort of individuals indicated that the MLPA method provided more accurate genotyping. The MLPA method showed an unexpected range of CNV at the KIR locus. Segregation analyses in pedigrees previously genotyped by PCR-SSP confirmed the strength and accuracy of the MLPA method and helped to identify duplication events in the KIR gene cluster in one of these families.

Taken together, CNV at the KIR locus is extensive. The KIR MLPA assay can accurately determine an individual’s KIR genotype in a highly efficient manner, allowing routine KIR genotyping in transplantation programs and offering the opportunity to increase the success rates of transplantation or graft-versus-leukemia effects. Also, genotype-phenotype relations may be studied in greater detail to better understand the exact role of KIRs in health and disease.

## Results

### KIR Genotyping by MLPA Methodology

In designing the set of probes for the KIR MLPA we used the following principles. *First,* making use of publicly available databases (http://www.ebi.ac.uk/ipd/kir/), we selected single nucleotide polymorphisms (SNPs) in each KIR gene that selectively distinguish one gene from all others. In some cases two of those SNPs were combined together in one probe set that consists of three probe parts, to make the probe segregating between the KIR genes. This has previously been applied in the MLPA assay for the complement genes in the HLA class III region [20]. The KIR gene-specific SNPs were chosen such that all allelic variants would be recognized by the corresponding probe. *Secondly*, some specific, additional KIR variant sequences were selected to make a further distinction among the variants of a particular KIR gene. In this way, a probe-based distinction was possible between wild-type *KIR2DS4* and truncated variants of this gene (KIR2DS4*003-*010, *012, *013) [21]–[23]. Next, we designed our probes mainly inside exons of the selected gene sequences (Tables S1 and S2). One allelic variant of *KIR3DL1* (KIR3DL1*054) is not detected, because this allele contains a SNP at the specific probe-binding site.

Where possible, two separate independent probes per KIR gene were included in the assay as an internal check on the presence or absence of any of the genes at the KIR gene cluster. The latter is particularly important for excluding putative false-negative results due to SNPs that have not been documented yet. To avoid, on the one hand, possible competition between probes for the same KIR gene and on the other hand to accommodate all probe amplification products in the same assay, we divided the probes over three different probe mixes (Table S3).

### Accuracy of the KIR MLPA Assay

The MLPA method was subsequently performed to genotype 120 Caucasian individuals (Table 1). The occurrence frequency of KIR genes in our study population of control individuals was similar to the frequency reported in the “Allele Frequency Net Database” (http://www.allelefrequencies.net/kir6002a.asp; update November 2011 [24]) (Table 1), hence lending credibility to the MLPA-derived genotyping of KIR genes.

**Table 1:** KIR genotype frequency as determined by MLPA, compared with data from the allele frequency database and determination by PCR-SSP.

KIR Gene	Allele frequency		MLPA *vs* PCR-SSP
	MLPA	Database*	
**2DL1**	95.8%	87.8–100%	99.2%
**2DL2**	53.3%	32–67.9%	100%
**2DL3**	87.5%	84–97%	100%
**2DL4**	100.0%	99–100%	100%
**2DL5**	56.3%	41.1–67%	98.3%
**2DS1**	37.5%	27–54.5%	93.3%
**2DS2**	53.8%	40–63%	100%
**2DS3**	31.7%	18.7–41%	100%
**2DS4all**	96.6%	83.1–98.8%	100%
**2DS4wt**	39.2%	33–49.6%	100%
**2DS4trunc**	81.7%	44.2–89%	100%**
**2DS5**	33.3%	20.8–43%	100%
**2DP1**	95.8%	94–100%	99.2%
**3DL1**	96.7%	89–98.5%	100%
**3DL2**	100.0%	100.0%	100%
**3DL3**	100.0%	100.0%	100%
**3DS1**	41.7%	27–63%	100%
**3DP1**	100.0%	97–100%	100%

n = 120; * Range of Caucasoid populations www.allelefrequencies.net/kir6002a.asp;

** SSP data available for 25 donors.

PCR with sequence-specific primers (SSP) was performed routinely in parallel to evaluate the accuracy of the MLPA assay by comparing the results in the same cohort of 120 individuals (Table 1). Upon comparison, the datasets of both assays matched for 99.5%. Of the 17 KIR genes per donor typed by both methods, in these 120 individuals 12 individuals showed a mismatch in a single KIR gene.

**Figure 1:**
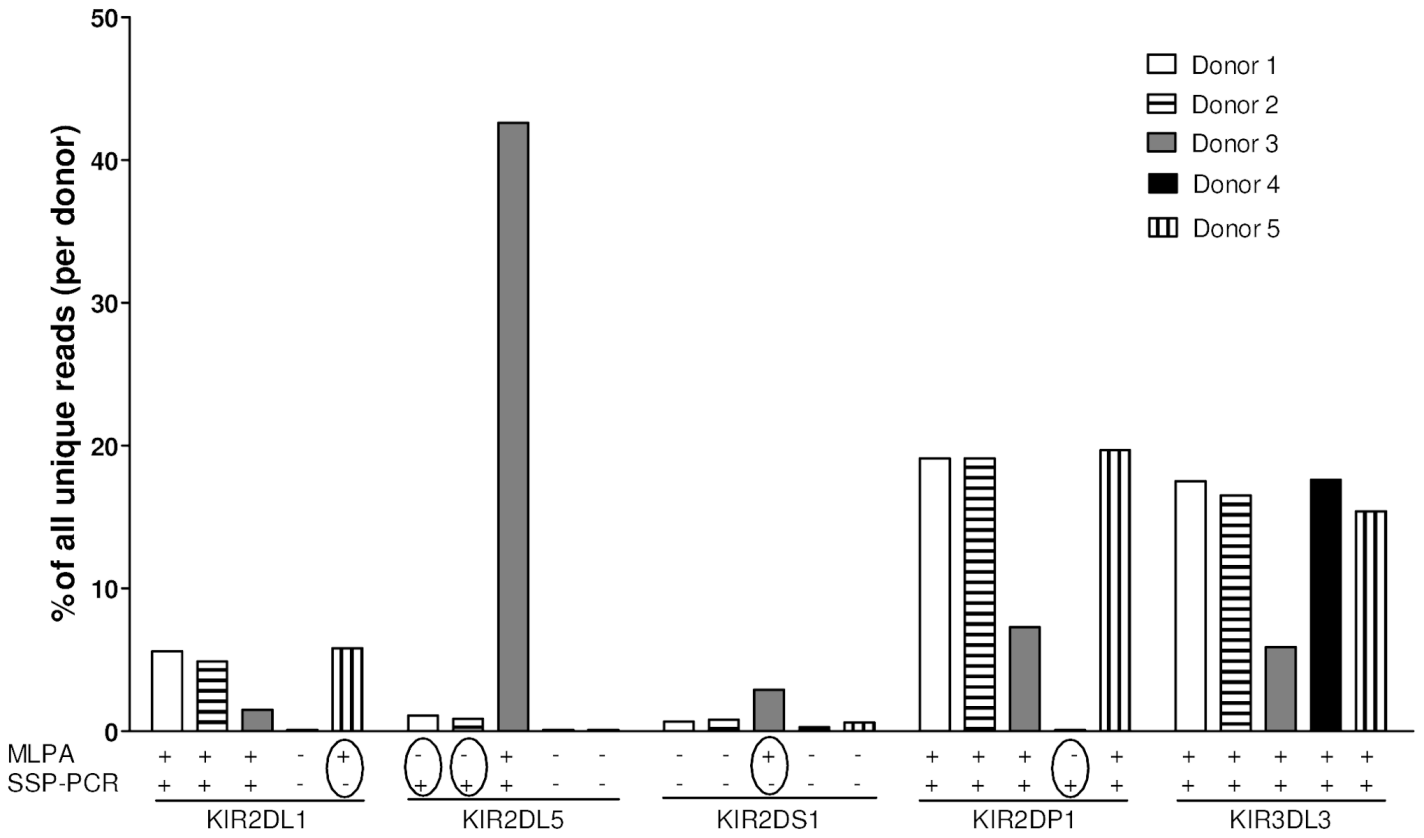
Validation of the accuracy of genotyping with MLPA by next-generation sequencing (NGS). Five of the genomes with mismatches between MLPA and PCR-SSP were analyzed by NGS. The percentage of reads that aligned against a reference for a KIR gene gives an impression about the presence of the KIR gene in that specific donor. Circles indicate the mismatches found between MLPA and PCR-SSP. *KIR3DL3* is a control gene as it is present in all donors.

All KIR genes of 5 individuals were amplified by long-range PCR and sequenced by Next-generation sequencing (NGS) on a 454 FLX Roche Genome Sequencer to resolve these discrepancies (Figure 1). In all cases, the methodological discrepancies appeared to be due to false-positive or false-negative PCR reactions in the PCR-SSP. Upon re-evaluation of the PCR-SSP data, some explanations for the mismatches were found. For one, *KIR2DS1* is represented by a long 1800 base-pair PCR product, leading to some false-negative results for this KIR gene in the PCR-SSP, due to insufficient amplification. Also, the particular PCR product detected for *KIR2DP1* appeared to be too long, leading to an aspecific product and a false-positive result for *KIR2DP1* in the PCR-SSP. From these results we conclude that the KIR MLPA method performed with a higher degree of accuracy than the PCR-SSP method.

### Quantification of KIR CNV with a Calibrator

**Figure 2:**
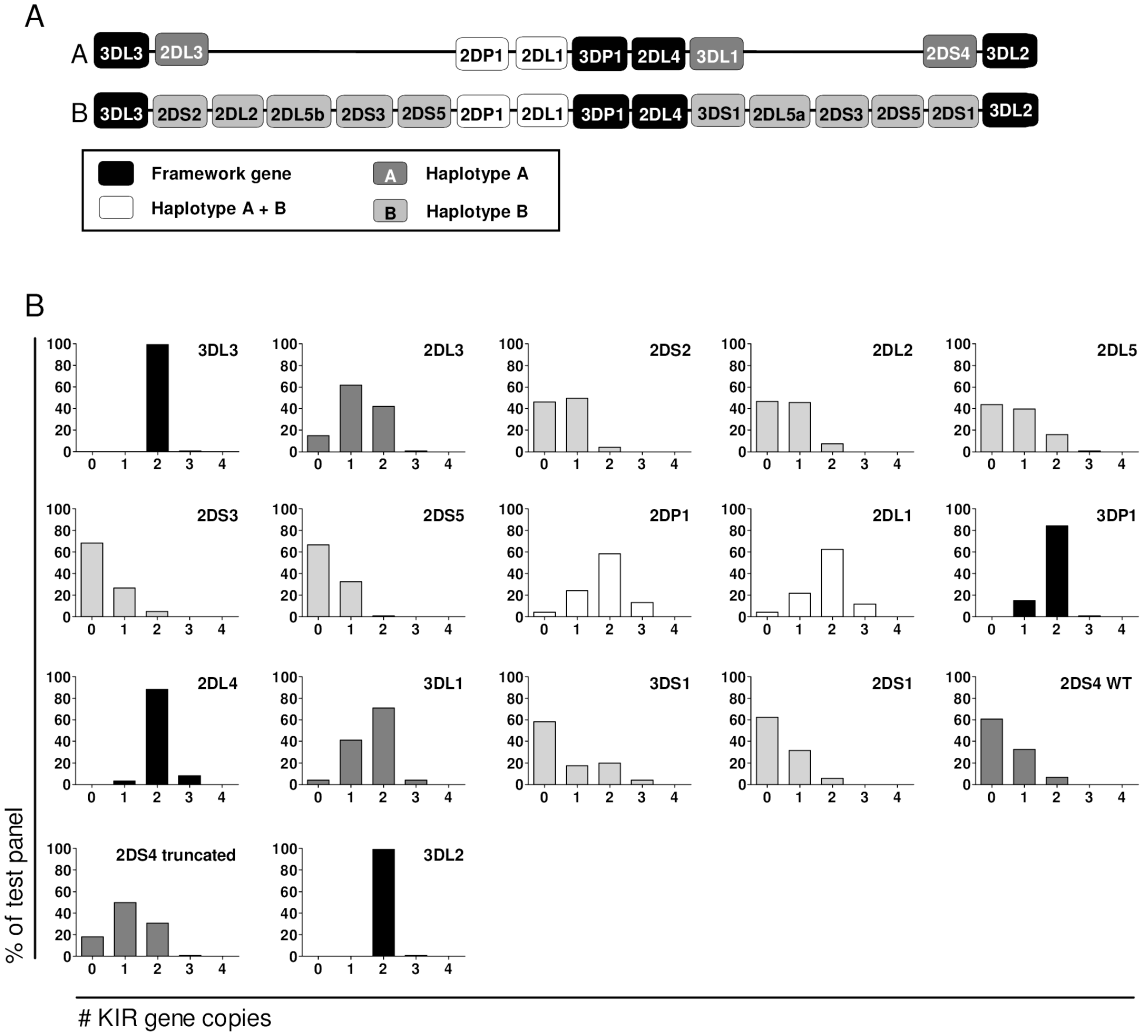
KIR gene copy number determination of a large cohort by MLPA. (A) Overview of the Killer Immunoglobulin-like Receptor gene family. The genes are arranged in two haplotypes (A and B), which both always contain the framework genes as depicted in black. Both haplotypes may or may not contain the genes in white. The genes depicted in grey are supposedly found only on haplotype A or B. (B) KIR gene copy numbers were determined for 120 individuals by MLPA. Shown is a graphical representation of the percentages of donors with 0, 1, 2, 3 or more than 3 copies of each KIR gene in their genome.

The proposed haplotypes (Figure 2A) contain a variable number of KIR genes within each individual’s genome, leading to gene copy number variation (CNV). Within each genome particular genes may be absent or present once, twice and sometimes even more than twice [12], [14]–[16], [25]. Our previous experience with the MLPA methodology had already indicated that this technique allows a reliable assessment of the gene copy number in the human genome [18], [20]. However, the MLPA also has some disadvantages, one of which is the use of a reference DNA sample that is employed to determine the gene copy number and to reduce inter-assay variation. Since the KIR genotype of this reference sample is unknown, this does not allow an accurate determination of individual KIR gene CNV. To circumvent this problem we created a synthetic control as an internal calibrator for our KIR MLPA method. This internal standard consists of a DNA vector that includes the sequences of all of the probe-binding sites once. The gene-assembly method, as previously described by Stemmer and co-workers, was adapted to create the KIR calibrator [26].

With this calibrator we analyzed the cohort of 120 individuals for CNV (Figure 2B, Tables S4 and S6). According to our previous experience the number of 0, 1, 2, 3 and >3 genes can be discriminated in a highly reproducible manner. Several results should be noted. First, the framework genes *KIR3DL2* and *KIR3DL3* were represented by 2 copies in most individuals, which fits with the current A and B haplotype distribution system [13]. However, the other two framework genes *KIR3DP1* and *KIR2DL4*, separating the two blocks of genes in each haplotype (Figure 2A), have been deleted or duplicated in a small percentage of individuals. *KIR2DL4* occurs 2 times in 88% of the individuals, which suggests that translocation events have taken place in 12% of the individuals. The same seems true for *KIR3DP1*, which occurs 2 times in 84% of the individuals. Furthermore, we observed variation in gene copy numbers for *KIR2DL1*, *KIR2DP1*, *KIR2DL3* and *KIR3DL1*. The *KIR2DL3* and *KIR3DL1* genes, which are considered to be specific for the so-called A haplotype [12], occur in relatively high numbers, with >70% of individuals carrying 2 or 3 copies. Finally, several of the activating genes of the so-called B haplotype [12], such as *KIR2DS1*, *KIR2DS2* and *KIR2DS5*, are rarely seen in 2 or more copies within the genome of the population tested. Within the tested population, the average number of copies per inhibiting receptor was 1.2 (not including the framework genes), while the average number of copies for activating receptors was 0.5.

### Quantification of CNV by MLPA Validated by Sequencing

To validate whether our MLPA could determine CNV accurately, we needed an independent assay that could quantify the number of copies of the same gene or the number of the same gene fragments. For this purpose, we used the presence of short tandem repeats (STRs) in the KIR gene locus. STRs are short sequences that have been duplicated multiple times, resulting in stretches of conserved repeated sequences. In KIR genes, several STR regions exist, one of which is a repeat of 4 base pairs, AGAT, located in intron 4 of most KIR genes [25], [27], [28]. Closer examination of this region showed that each KIR gene contains an invariable number of repeats, with the exception of *KIR2DL4* and *KIR2DL5*, which lack the typical intron 4 of the other KIR genes. Within this STR region single nucleotide polymorphisms (SNPs) are present, which allows for identification of the individual KIR genes even if the number of the repeats is equal.

It is impossible to determine CNV of the KIR genes by these STRs by MLPA methodology. Apart from the many technical issues with respect to the design of such probes, these STRs may not only be present within the KIR gene cluster but might occur more widely throughout the human genome. However, it is possible to use the information about STRs within the KIR gene cluster for an independent validation of KIR CNV quantification obtained by the MLPA method. NGS enabled specific STR detection to determine the presence or absence of KIR genes (except *KIR2DL4* and *KIR2DL5*). CNV can be quantified by determining the coverage of the amplified product, *i.e.* the relative occurrence of a certain STR length and specific sequence, as a signature of the corresponding KIR gene.

**Figure 3:**
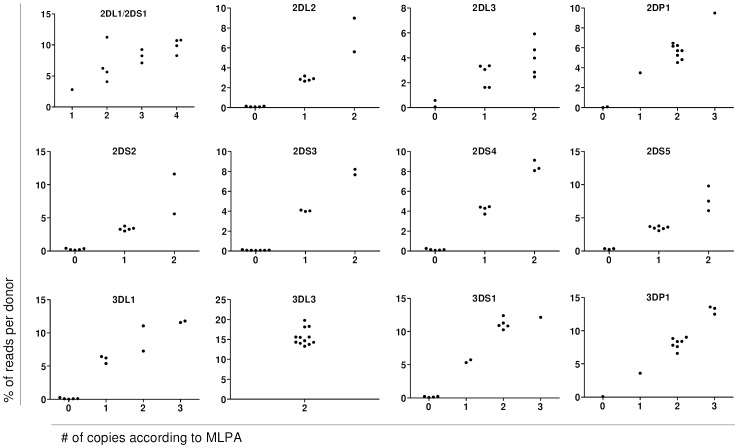
Validation of the KIR MLPA method with an independent short tandem repeat (STR) assay. A primer set was used to amplify a stretch of repeats that occurs in intron 4 of most KIR genes. Next-generation sequencing (NGS) was used to sequence the amplification products based on both specific sequence and number of repeats. The graphs show the percentage of reads in NGS, aligned against the reference sequence of that KIR, in donors with a certain number of copies of that gene as determined by MLPA. The number of reads of framework gene *KIR3DL2* was used as a reference to adjust for the total number of genes per donor. *KIR2DL1* and *KIR2DS1* have the same number of STR and no SNPs to distinguish one from the other and have therefore been combined. Several SNPs within the repeat region of *KIR2DL3* and *KIR3DL3* confirm the copy numbers as found with the MLPA method.

Hence, to evaluate the accuracy of CNV determination in the KIR MLPA assay, primers were designed to amplify these repeat stretches in 12 samples. The PCR products were specific for each KIR gene because of the sequence and length (ranging from 390 to 450 bp; Table S5). Subtle sequence differences allow for a distinction between KIR genes with the same number of repeats, except for *KIR2DL1* and *KIR2DS1*, which are inseparable in this region. NGS yielded information about the copy number of the KIR-specific STRs of these 12 samples, which corresponded 100% with the MLPA test results (Figure 3). The STR assay thus confirmed the MLPA data for the KIR genes that can be detected with this assay.

### Copy Number Variation Validated within CEPH Pedigrees

**Figure 4:**
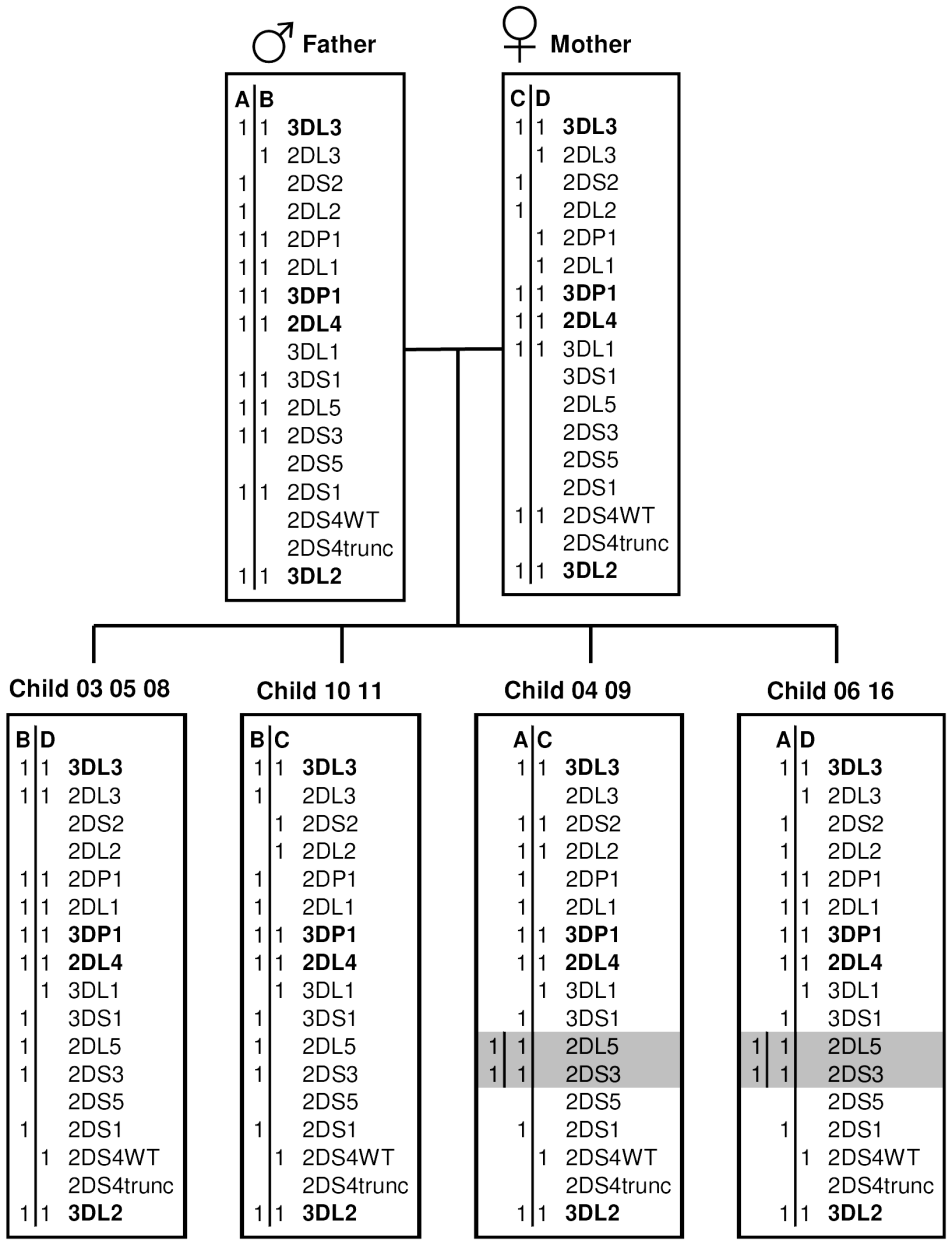
KIR gene pedigree analysis of a Centre d’Etude du Polymorphisme Humaine family by KIR-MLPA. Children 04, 06, 09 and 16 of family 1347 show a translocation event involving *KIR2DL5* and *KIR2DS3*.

The human genome diversity cell line panel (HGDP) and Centre d’Etude du Polymorphisme Humaine (CEPH) bank contain a large number of cultured lymphoblastoid cell lines (LCLs) that are available for family segregation analyses and genome research (http://www.cephb.fr/en/hgdp/diversity.php). We typed the KIR genome of two generations of 3 CEPH families and a family from Israel. Family segregation analysis functions as a third form of validation, because the CEPH families have been extensively typed by other groups with PCR-SSP [16], [29]. These families were typed in detail by KIR MLPA because of CNV and the indication of duplication events. Using the KIR gene calibrator as our reference sample, we determined the CNV for all the KIR genes of both parents and some or all children in these families and mapped possible inheritance patterns (Figure 4, Figures S1, S2 and S3). For all families tested, the genes of all genotyped children were traced back to the two alleles of both parents.

Interestingly, in one family (Figure 4) we witnessed a cross-over of part of allele B to allele A. The father has 2 copies each of *KIR2DL5* and *KIR2DS3*, while the mother has none. These genes are present twice in the children that inherited the A allele of the father. As the siblings that received the B allele only have one copy of each gene, this excludes the possibility that the genes were already duplicated in the father. This duplication event was confirmed with the STR assay described above for *KIR2DS3* and with a qPCR on DNA for *KIR2DL5* and *KIR2DS3* (Figure S4). The mother of family 1416 (Figure S1) contained a duplication of *KIR3DP1* and *KIR2DL4*, which was subsequently detected in three of the children. These data confirm the results of Martin and co-workers [14] for the same family, who established a crossing-over event of part of a haplotype between *KIR2DL5* and *KIR3DP1*. The translocation event described by Traherne and co-workers [16] in family 1413 (Figure S2) can also be detected by the MLPA method. In addition, when testing another series of untyped control pedigrees we picked up a family with a lack of the framework genes *KIR2DL4* and *KIR3DP1* in all three children (Figure S3), a phenomenon that was previously reported to occur only rarely [30]. These observations lead us to conclude that the KIR gene cluster is subject to more translocation events than is generally assumed.

## Materials and Methods

### Ethics Statement

The study was approved by the Institutional Medical Ethics Committee of the Academic Medical Center in Amsterdam and was performed in accordance with the Declaration of Helsinki. Participants provided their written informed consent to participate in this study.

### DNA Isolation

DNA was purified from 120 healthy donors with a QIAgen Blood Kit (Qiagen, Venlo, The Netherlands) according to the manufacturer’s protocol.

### Multiplex Ligation-dependent Probe Amplification

The KIR MLPA is based on the method described by Breunis et al. [18]. In short, 100 ng of DNA was denatured at 98°C for 5 min in a thermocycler with heated lid and cooled to 25°C. Three µl of probe mixture, containing KIR-specific probes (Table S1), control probes (Table S2) and competitor probes, was added and denatured at 95°C for 1 min, then hybridized at 60°C for at least 16 hours. The mixture was cooled to 54°C before addition of 32 µl of Ligase 65 mixture (MRC Holland, Amsterdam, The Netherlands). Ligation took place at 54°C for 15 min, deactivation at 98°C for 5 min, and the mixture was cooled to 4°C. Bound probes were amplified by PCR: 10 µl of ligation mixture was added to 5 µl of Accuprime Taq Buffer, 0.4 µl of Accuprime Taq enzymes (AccuPrime Taq DNA Polymerase High Fidelity kit, Life Technologies, Carlsbad, CA, USA) 1.5 µl of 50 mM MgSO_4_ and 1.5 µl of a single primer pair of which one primer was labeled with a FAM group. PCR conditions included 36 cycles of 30 sec denaturation at 95°C, 30 sec annealing at 60°C and 1 min elongation at 68°C, followed by 20 min elongation at 68°C. Fluorescently labeled products were thus created, allowing for fragment analysis by capillary electrophoresis. 1.5 µl of the PCR reaction was mixed with 9 µl of deionized formamide and 1 µl of Promega Internal Lane Standard 60–600 (Promega, Madison, WI, USA) and incubated at 90°C for 10 min before product separation on an ABI-3130XL (Applied Biosystems by Life Technologies). Fragment analysis was done with the program GeneMarker (version 1.90, SoftGenetics, State College, PA, USA).

### Next-Generation Sequencing

For whole KIR genome sequencing, a long-range PCR was performed of each donor. Several primers were designed that amplified the whole KIR genome; FW universal (5′-gccaaataacatcctgtgcgctgctcagct-3′), FW 3DL3 (5′-ctcacaacatcctgtgtgctgctaactga-3′), FW 2DL4 (5′-cacatcgtctgcaccggtcagtcgagccga-3′), REV universal (5′-ttggagaggtgggcaggggtcaagtg-3′) and REV 3DP1 (5′-ctccatctgagggtcccctgaatgtg-3′). The PCR reaction was performed with 500 µM dNTPs, 1x PCR Buffer 2 (Expand Long Template PCR system, Roche Diagnostics, 454 Life Sciences, Branford, CT, USA), 300 µM of both primers and 3.75 U polymerase enzyme mix (Expand Long Template PCR system, Roche Diagnostics). The PCR program consisted of an initial denaturation of 94°C followed by 10 cycles of 10 sec at 94°C, 30 sec at 60°C and 12 min at 68°C, then another 20 cycles in which the denaturation at 94°C was extended to 15 sec, followed by final elongation of 7 min at 68°C. The resulting PCR products were up to 17 kb in length.

The fragments were sequenced on a 454 FLX Roche Genome Sequencer (Roche Diagnostics, 454 Life Sciences, Branford, CT, USA), with the Shotgun Library method. ReferenceMapper software (Roche Diagnostics) was used to align the reads to a reference sequence.

### Synthesis of the KIR-MLPA Calibrator

The KIR-MLPA calibrator was created with an assembly method adjusted from Stemmer *et al.* [26]. The intended synthetic product was divided in six separate sequences. Each sequence was cut up into small oligodeoxyribonucleotides of 60 base pairs that covered either the sense or the anti-sense strand with a 20-base pair overlap of each strand. These oligo’s were synthesized by Life Technologies (Carlsbad, CA, USA). Multiple 60-mers were fused together in a PCR reaction: 2 µM oligo’s, 0.2 µl of Accuprime Taq polymerase, 0.4 µl of Accuprime Pfx polymerase, 2 µl of Buffer I (Life Technologies) and de-ionized water were combined to a volume of 20 µl. This reaction contained a denaturation step at 94°C for 15 sec, then 55 cycles of 30 sec at 94°C, 30 sec at 52°C, 60 sec at 68°C. Subsequent amplification of 1 µl of this product with 5 µl of the two outer oligo’s of each synthesized sequence, 0.5 µl of Accuprime Taq polymerase, 1 µl of Accuprime Pfx polymerase, 5 µl of Buffer I and de-ionized water up to 50 µl. This second PCR program included 24 cycles of 30 sec at 94°C, 30 sec at 52°C, 90 sec at 68°C.

With this method, products of around 680 base pairs were synthesized. For larger sequences, 1 µl of two 680-mers were pooled together (with an overlap of 180 bp) and subjected to another round of PCR. Genes larger than 1180 bp were synthesized in multiple steps of these assembly rounds, until the right size was achieved.

These products were sequenced by Sanger sequencing, to check for mutations. The mutations were mutated back to their original sequence by means of the Quickchange protocol (Stratagene, La Jolla, CA, USA) as described by the manufacturer. Transformation in *E. coli* and subsequent sequencing of the plasmids was used to check the final product.

### Copy Number Determination with the KIR-MLPA Calibrator

The calibrator was added in a comparable number of molecules as present in an average human genome of 100 ng. Because the calibrator has a length of approximately 11 kb, there is relatively little DNA present in each sample. To prevent the DNA from sticking to the plastic storage tube, we used 400 ng/ml herring sperm DNA as a decoy. Also, LoBind tubes (Eppendorf, Nijmegen, The Netherlands) were used to prevent the DNA from sticking to the plastic. The calibrator served as a reference and was run in parallel to the test samples in every MLPA run as an extra sample. To minimize variation within one experiment, one calibrator was present for every 7 samples.

Analysis was performed with GeneMarker software (Softgenetics). The amounts of amplification product that were measured for all calibrator samples within one run were averaged and used as one reference sample. The software calculated a ratio for the peak height or area of each test sample against the corresponding (averaged) calibrator peak. The peak pattern of the calibrator represented 2 copies of each KIR gene, after normalization with the software. Therefore, a ratio of 1 means that 2 copies of this gene were present in the test sample. The control probes provided a quality control for each test, as each gene targeted by these probes is present twice in each individual.

Not all probes in our MLPA gave a good approximation of the copy number. The probes that were used for copy number determination are listed in Table S4. When multiple probe sets were used for copy number determination, an average was calculated of all ratios. We apply as a rule of thumb that an (average) ratio of 0.25–0.75 represents 1 copy, 0.75–1.25 represents 2 copies, 1.25–1.75 represents 3 copies and >1.75 represents 4 or more copies. For border cases multiple runs were taken into account.

### PCR-SSP

The polymerase chain reaction with sequence-specific primers (PCR-SSP) was performed as previously described [4], [12].

### Sanger Sequencing

Sequence analysis was performed according to the manufacturer’s protocol of the BigDyeTerminator cycle sequencing kit on an ABI-3130XL (Applied Biosystems by Life Technologies).

### Short Tandem Repeat Assay

Primers were designed around the short tandem repeat region in intron 4, present in all intron 4 containing KIR genes (sense 5′-ccaaagagaactagagagaccgagaggc-3′, 5′ccaaatagaactagagagactgagaggc-3′, 5′-ccaaagagagctagagagaccgagaggc-3′, 5′-ccaaaagggaactagagagactgagaggc-3′ and anti-sense 5′-tgtgtccttgtgtcctgttcataactttctgc-3′, 5′-tgtgtccttgtgtcctgtccataactttctgc-3′, 5′-tgtgtccttctgtcttgctcataactttctgc-3′, 5′-tgtgtccttctgtcttgttcataactttctgc-3′, 5′-tgtgtccttgtgtcccgttcataactttctgc-3′). One PCR reaction amplified all short tandem repeat regions (Table S5): 5 µl of 20 ng/µl DNA was mixed with 12.6 µl of de-ionized water, 3 µl of SalsaBuffer (MRC Holland), 1.2 µl of 10 mM dNTP, 1 µl of each primer mix with a 10 pmol/primer/µl concentration. 0.4 µl of Taq Start Antibody (Clontech, Saint-Germain-en-Laye, France) and 0.8 µl of Salsa DNA polymerase (MRC Holland) were combined on ice and added to the DNA mix. The PCR program consisted of 2 min denaturation at 94°C, followed by 36 cycles of 15 sec denaturation at 95°C, 1 min annealing at 58°C and 5 min elongation at 68 °C. The final step was an elongation at 68°C of 20 min.

The amplified fragments were processed on a 454 FLX Roche Genome Sequencer, by the Shotgun Library method, followed by analysis with ReferenceMapper. The percentage of reads per donor per KIR gene was normalized against the framework gene *KIR3DL2* to compensate for the difference in total gene copy numbers between donors. The percentage of reads that were found per KIR gene represented a relative number of copies present of that gene. A reference sequence was used to sort the reads, based upon the sequences within the STR region of the KIRs, available from the IPD KIR database: http://www.ebi.ac.uk/ipd/kir/(Release 2.4.0, 15 April 2011).

### Quantitative DNA PCR

A quantitative PCR on DNA was designed to validate gene copy number determination by MLPA. Primers were designed for *KIR2DL5* (sense 5′-gctggctccacatcctcgtt-3′ and anti-sense 3′-cccaagacgagagcgactca-3′) and for *KIR2DS3* (sense 5′-ccaagatcagcaagtgtgggttt-3′ and anti-sense 5′-cttggcaggaggtatgaactcaa-3′). The qPCR was performed as described earlier [31]. The relative ratio is a result of the absolute numbers from the KIR genes compared to the absolute numbers of a PCR on exon 8 of the Cytochrome *b_558_* beta chain, gene (*CYBB*) (Sense: 5′-atgtcaaatatttaagcaagcctac-3′ and anti-sense 5′-acttgtccatgatatagttagacac-3′). The *CYBB* numbers are corrected for sex, as this gene is present on the X-chromosome.

## Discussion

In this study we have demonstrated the presence of extensive CNV at the KIR gene cluster. To determine KIR genotype and copy number, we designed a novel MLPA assay, using a synthetic calibrator as an internal reference. We found more gene copies for inhibiting than for activating KIRs. Similar gene copy variation was unexpectedly found in a number of the framework genes. This suggests either a more complex role for these supposed framework genes in the haplotype system than assumed until now, and/or crossing-over events, deletions and duplications in the KIR gene cluster occur at a far higher extent than previously thought. Because more variation exists in the middle framework gene block containing *KIR2DL4* and *KIR3DP1* than in the outer framework genes, *KIR3DL2* and *KIR3DL3*, frequent duplications within the KIR gene cluster seem likely. Although it has been shown before that duplications, crossing-over events and deletions in the KIR gene cluster can occur [14], [16], the frequency of these events has never been evaluated in detail.

To confirm our findings, next-generation sequencing was applied to validate our assay for KIR genotyping. No false-positive or false-negative genotyping was apparent when checked by 454 sequencing. An STR assay was used as an independent technique to measure CNV in the KIR gene cluster. This assay confirmed the copy numbers as found by the MLPA method for those KIRs that could be identified with the STR assay.

The analysis of hereditary patterns of KIRs in several families supports the importance of copy number determination. Using the MLPA assay, we detected several independent duplication/deletion events in previously typed CEPH families and in one additional family. One of these duplications represents a previously unreported event. To our knowledge, there are no analytical techniques available to easily detect these events at the KIR gene cluster, explaining the fact that one of these events went undetected in these ‘standard’ pedigrees until now.

The MLPA assay is designed to detect the presence of entire genes, based on the current knowledge. Novel genes and hybrid genes are not specifically targeted when using this technique. From our 454-sequencing data and the probe analysis of our MLPA method, we know that the hybrid gene *KIR2DL5A/3DP1* [14] (3DP1*004) is detected in our MLPA method as *KIR3DP1*. The hybrid gene *KIR2DL3/2DP1* [16] is not detected at all and the hybrid gene *KIR2DL1/2DS1* [16] is detected by both the *KIR2DL1* and *KIR2DS1* probes. As the first two genes are most likely pseudogenes, only the latter causes concern for proper detection with the current MLPA assay. The hybrid gene *KIR2DL1/2DS1* likely resembles *KIR2DS1* the most, as fusion of the two sequences occurs before the first transcribed domain, which would result in a false-positive *KIR2DL1* scoring by the KIR MLPA technique. Nevertheless, the frequency of this hybrid gene is expected to be very low, as was reported by Traherne et al. [16]. In addition to the 2 families in which this gene was originally found, the hybrid gene was detected in only 3 (non-Caucasian) individuals of 1214 samples from various ethnic origins. To the best of our knowledge, no protein expression has been confirmed to date.

Also, specific allelic variants were not separately targeted. There are several KIR genes with allelic variants that are not expressed. Specific targeting of these allelic variants could give an even more accurate KIR genotype. Although the MLPA assay allows further expansion of probe sets when required, we have to consider the additional relative value of such probes to detect rare allelic variants because of the low frequency in cohort studies.

From an evolutionary perspective, one would expect an immunological effect of CNV at the KIR gene cluster, as shown for other immune receptors [18]. In fact, studies have been published that indicate a relationship between CNV and transcription levels for several KIRs [32], and also the relevance of CNV in the KIR gene cluster in relation to certain diseases has been suggested for *KIR3DL1* and *KIR3DS1* [15], [17], [33], [34].

An NK cell resides in a delicate balance between activated and inhibited state. Expression seems to be regulated in a clonal fashion, in which each cell expresses its own selection of receptors. From this notion, we can hypothesize that it is also a selection of several receptors that decides cell fate during disease. Indeed, the presence of some activating KIR-HLA pairs in combination with a lack of inhibitory receptor pairs has been associated with an increased risk of developing certain autoimmune diseases like Diabetes type 1 and Ankylosing Spondylitis [35], [36]. Also, the total number of activating KIR genes might influence NK cell effector function, as has been suggested for CMV reactivation during HSCT [37], even though we still do not know the nature of the ligands for most of these activating KIRs. The use of our KIR MLPA creates interesting extra dimensions to the analysis of such data panels, adding information on the presence or absence of CNV.

KIRs are mostly expressed by NK cells, which have a role in cancer surveillance and host defense against viral infections. A pivotal role for NK cells in human disease was shown for the first time in a rare severe immunodeficiency disease (SCID) patient who was able to clear CMV infection in the absence of T cells [38]. A similar situation can be encountered early after myeloablative HSCT, since donor-derived NK cells reconstitute earlier than T cells [5], [8], [39]. In experimental models and in clinical trials, NK cells have received much attention in transplantation settings during cancer treatment. NK cells have been demonstrated not to cause Graft-versus-host-disease (GVHD), even when alloreactive across major histocompatibility barriers [5], [40]. Unlike T cells that carry the risk of causing lethal GVHD, NK cells are safe as a form of cellular immunotherapy in the allogeneic HSCT setting. Indeed, alloreactive NK cells are exploited as mediators of graft-versus-leukemia (GVL) effects in T-cell-depleted haploidentical HSCT for acute myeloid leukemia following HSCT, or as potential cellular treatment in high-risk leukemia patients instead of HLA-identical HSCT [5], [8].

In summary, there is a clear need for simple but highly accurate tools to study KIR genotype-phenotype relations. We have therefore developed our current KIR MLPA assay for this purpose. Irrespective of the relative contribution of inhibitory or activating KIRs or lectins such as NKG2C or NKG2D in viral clearance and/or transplantation outcome [9], [39], realizing the potential role of CNV on KIR expression and functional activity as indicated in the present study, our current KIR MLPA assay will also enable us to relate disease severity to CNV in the KIR gene cluster in more detail on a larger scale. In addition, our MLPA assay for KIRs may be helpful in providing a rapid and efficient method for complete genotyping and at the same time provide data on CNV as a selection basis for donors in transplantation programs to improve the prognosis of graft survival and outcome of patients.

## Supporting Information

Figure S1 **KIR gene pedigree analysis of a Centre d’Etude du Polymorphisme Humaine family by KIR MLPA.** The mother of family 1416 has a duplication of two KIR genes on allele D, which was transferred to three of her genotyped children.
(PDF)

Figure S2 **KIR gene pedigree analysis of a Centre d’Etude du Polymorphisme Humaine family by KIR MLPA.** The father of family 1413 has only one copy of both *KIR2DL4* and *KIR3DP1*, which can be traced back to some of his children.
(PDF)

Figure S3 **KIR gene pedigree analysis of an Israeli family by KIR MLPA.** Both parents carry only one copy of the framework genes *KIR3DP1* and *KIR2DL4*, which results in a complete absence of those genes in all three children.
(PDF)

Figure S4 **Graphical representation of the quantitative PCR on DNA from Centre d’Etude du Polymorphisme Humaine family 1347 and some control donors.** (A) The relative product of KIR2DL5 compared to the occurrence of *CYBB* (corrected for sex). (B) The relative product of KIR2DS3 compared to the occurrence of *CYBB* (corrected for sex).
(PDF)

Table S1 **Overview probe sequences of the KIR MLPA technique.**
(PDF)

Table S2 **Overview control probe sequences of the KIR MLPA technique.**
(PDF)

Table S3 **KIR MLPA technique probe distribution over 3 mixes.**
(PDF)

Table S4 **Copy number determination by KIR MLPA technique.**
(PDF)

Table S5 **A sequence alignment of the short tandem repeat region in intron 4 of most KIRs.**
(PDF)

Table S6 **KIR gene copy number distribution in a cohort of healthy individuals.**
(PDF)
