# Supplementary figures and images for: Extensive Variation in Gene Copy Number at the Killer Immunoglobulin-Like Receptor Locus in Humans
Source: PLoS One. 2013 Jun 28;8(6):e67619. doi: 10.1371/journal.pone.0067619 (PMC3695908; doi:10.1371/journal.pone.0067619)

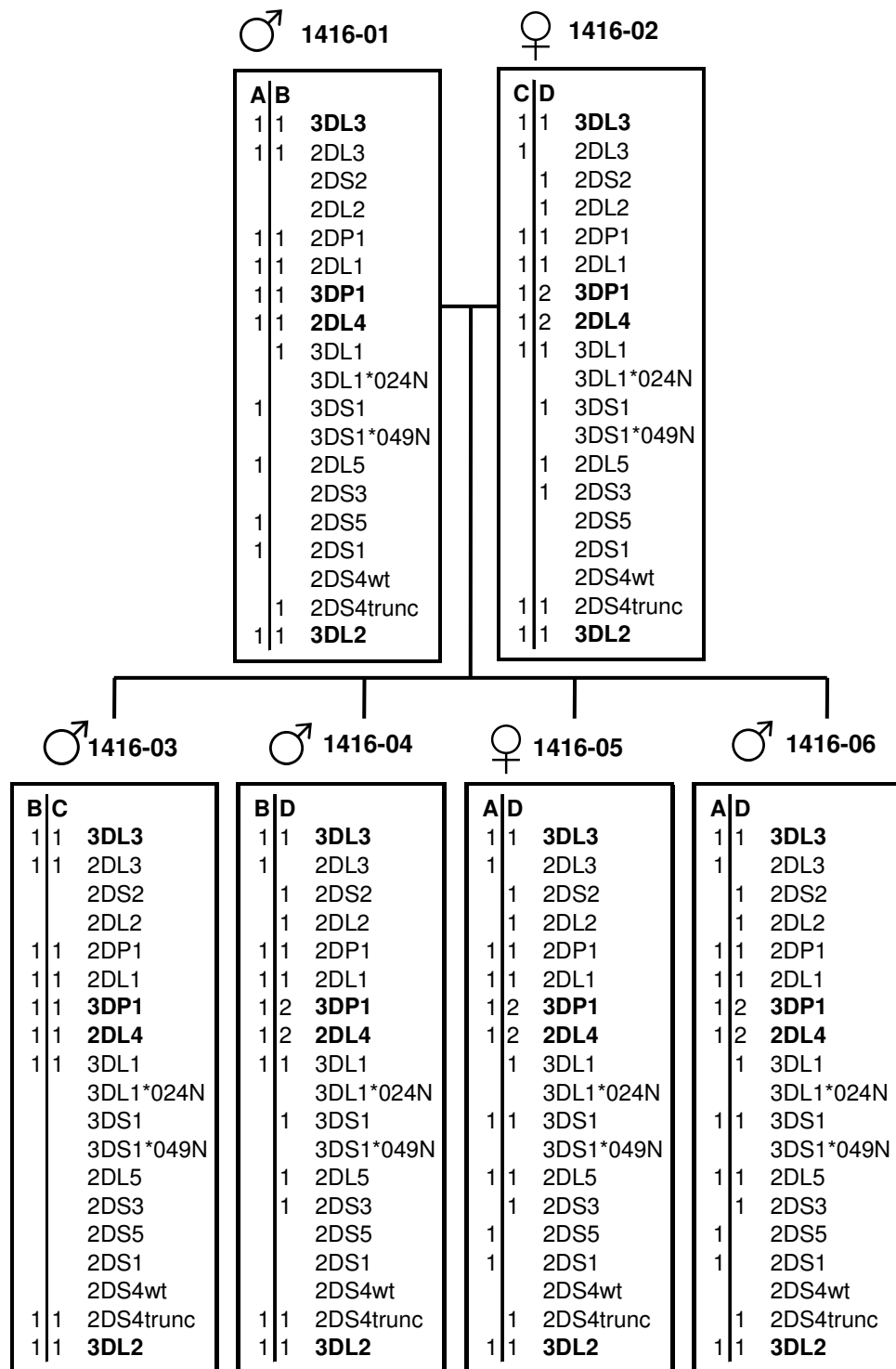

Supplement: Figure S1 — KIR gene pedigree analysis of a Centre d’Etude du Polymorphisme Humaine family by KIR MLPA. The mother of family 1416 has a duplication of two KIR genes on allele D, which was transferred to three of her genotyped children. (PDF) [file pone.0067619.s001.pdf]
