# Supplementary material for: Extensive Variation in Gene Copy Number at the Killer Immunoglobulin-Like Receptor Locus in Humans
Source: PLoS One. 2013 Jun 28;8(6):e67619. doi: 10.1371/journal.pone.0067619 (PMC3695908; doi:10.1371/journal.pone.0067619)
Supplement: Figure S2 — KIR gene pedigree analysis of a Centre d’Etude du Polymorphisme Humaine family by KIR MLPA. The father of family 1413 has only one copy of both KIR2DL4 and KIR3DP1, which can be traced back to some of his children. (PDF) [file pone.0067619.s002.pdf]

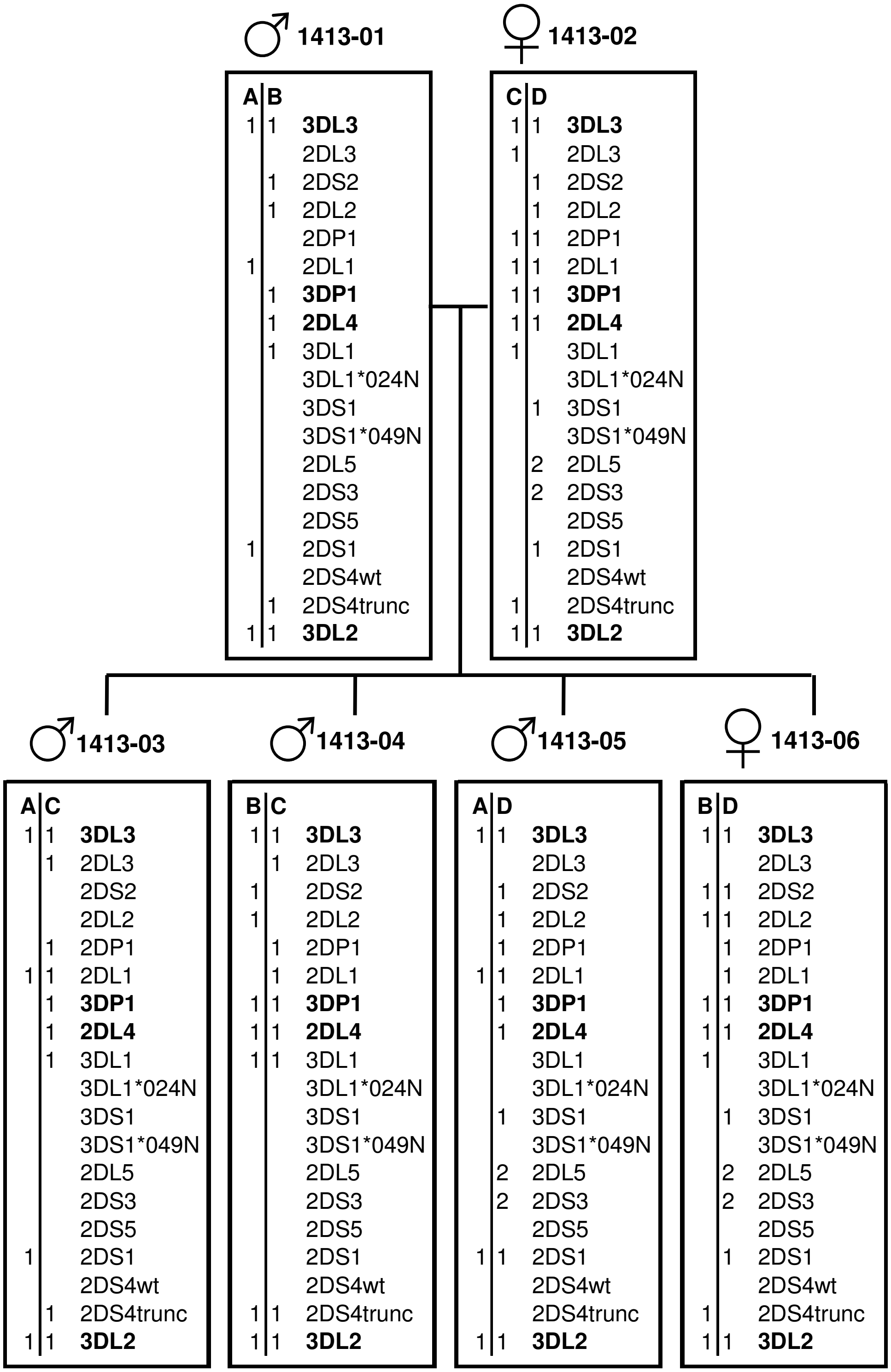

Figure S2

KIR gene pedigree analysis of a Centre d’Etude du Polymorphisme Humaine family by KIR MLPA. The father of family 1413 has only one copy of both *KIR2DL4* and *KIR3DP1*, which can be traced back to some of his children.
