# Supplementary material for: Extensive Variation in Gene Copy Number at the Killer Immunoglobulin-Like Receptor Locus in Humans
Source: PLoS One. 2013 Jun 28;8(6):e67619. doi: 10.1371/journal.pone.0067619 (PMC3695908; doi:10.1371/journal.pone.0067619)
Supplement: Figure S3 — KIR gene pedigree analysis of an Israeli family by KIR MLPA. Both parents carry only one copy of the framework genes KIR3DP1 and KIR2DL4, which results in a complete absence of those genes in all three children. (PDF) [file pone.0067619.s003.pdf]

♂ Father

A	B	
1	1	**3DL3**
		2DL3
1	1	2DS2
1	1	2DL2
		2DP1
		2DL1
1		**3DP1**
1		**2DL4**
1		3DL1
		3DS1
	1	2DL5
		2DS3
	1	2DS5
	1	2DS1
1		2DS4WT
		2DS4trunc
1	1	**3DL2**

♀ Mother

C	D	
1	1	**3DL3**
1		2DL3
	1	2DS2
	1	2DL2
1		2DP1
1		2DL1
1		**3DP1**
1		**2DL4**
		3DL1
1		3DS1
1	1	2DL5
		2DS3
1	1	2DS5
1	1	2DS1
		2DS4WT
		2DS4trunc
1	1	**3DL2**

♂ Child 1

B	D	
1	1	**3DL3**
		2DL3
1	1	2DS2
1	1	2DL2
		2DP1
		2DL1
		**3DP1**
		**2DL4**
		3DL1
		3DS1
1	1	2DL5
		2DS3
1	1	2DS5
1	1	2DS1
		2DS4WT
		2DS4trunc
1	1	**3DL2**

♂ Child 2

B	D	
1	1	**3DL3**
		2DL3
1	1	2DS2
1	1	2DL2
		2DP1
		2DL1
		**3DP1**
		**2DL4**
		3DL1
		3DS1
1	1	2DL5
		2DS3
1	1	2DS5
1	1	2DS1
		2DS4WT
		2DS4trunc
1	1	**3DL2**

♂ Child 3

B	D	
1	1	**3DL3**
		2DL3
1	1	2DS2
1	1	2DL2
		2DP1
		2DL1
		**3DP1**
		**2DL4**
		3DL1
		3DS1
1	1	2DL5
		2DS3
1	1	2DS5
1	1	2DS1
		2DS4WT
		2DS4trunc
1	1	**3DL2**

Figure S3

KIR gene pedigree analysis of an Israeli family by KIR MLPA. Both parents carry only one copy of the framework genes *KIR3DP1* and *KIR2DL4*, which results in a complete absence of those genes in all three children.
