# Supplementary material for: Extensive Variation in Gene Copy Number at the Killer Immunoglobulin-Like Receptor Locus in Humans
Source: PLoS One. 2013 Jun 28;8(6):e67619. doi: 10.1371/journal.pone.0067619 (PMC3695908; doi:10.1371/journal.pone.0067619)
Supplement: Figure S4 — Graphical representation of the quantitative PCR on DNA from Centre d’Etude du Polymorphisme Humaine family 1347 and some control donors. (A) The relative product of KIR2DL5 compared to the occurrence of CYBB (corrected for sex). (B) The relative product of KIR2DS3 compared to the occurrence of CYBB (corrected for sex). (PDF) [file pone.0067619.s004.pdf]

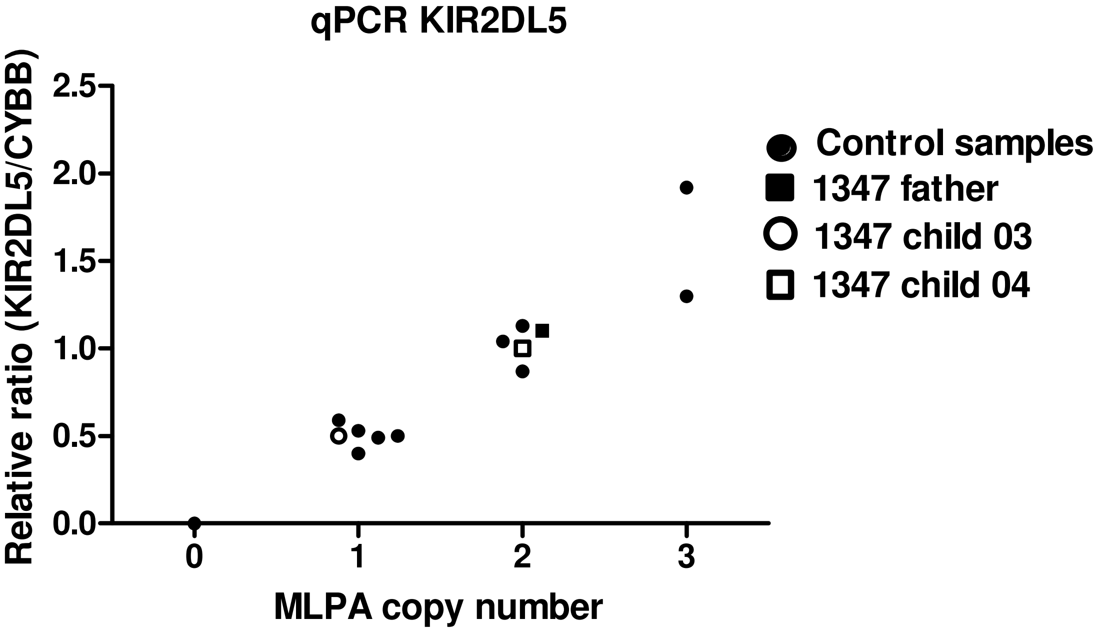

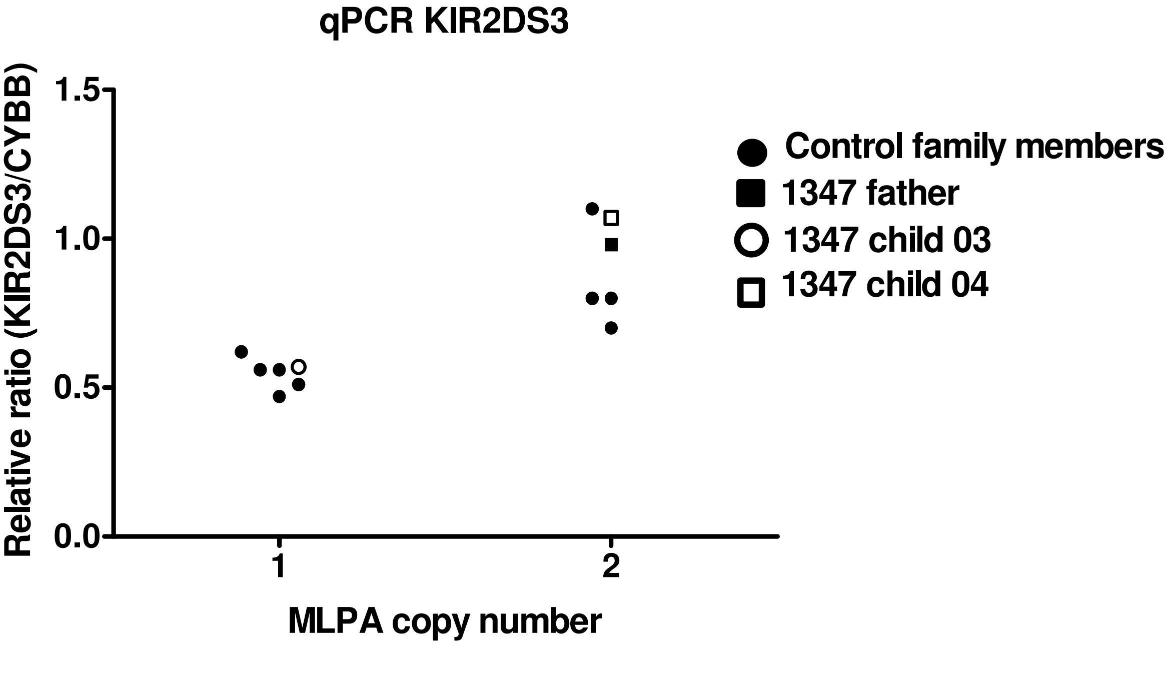

Figure S4

Graphical representation of the quantitative PCR on DNA from Centre d'Etude du Polymorphisme Humaine family 1347 and some control donors. (A) The relative product of KIR2DL5 compared to the occurrence of *CYBB* (corrected for sex). (B) The relative product of KIR2DS3 compared to the occurrence of *CYBB* (corrected for sex).
