# Supplementary material for: Extensive Variation in Gene Copy Number at the Killer Immunoglobulin-Like Receptor Locus in Humans
Source: PLoS One. 2013 Jun 28;8(6):e67619. doi: 10.1371/journal.pone.0067619 (PMC3695908; doi:10.1371/journal.pone.0067619)
Supplement: Table S1 — Overview probe sequences of the KIR MLPA technique. (PDF) [file pone.0067619.s005.pdf]

Table S1
Overview probe sequences of the KIR MLPA technique.

Probe	Sequence (5'-3')
2DL1 - #1 left	GGGTTCCCTAAGGGTTGGACTACCCCATCGCTCTTCATGCTGGATCATTCACTCTGCATCCCAATGACAATG
2DL1 - #1 middle	AGAAGAAAGTCTGGACACTCTCACCTATGATCACGATGTCCAGAGGGTCACTGGGAGCTGACAC
2DL1 - #1 right	CTGATAGGGGGAGTGAGTAACAGAACCGTAGTCTAGATTGGATCTTGCTGGCAC
2DL1 - #2 left	GGGTTCCCTAAGGGTTGGACATCCTGTGCGCTGCTGAGCTGAGCTCG
2DL1 - #2 right	GTCGCGGCTGCCTGTCTGCTCCGGCAGTCTAGATTGGATCTTGCTGGCAC
2DL2 - #1 left	GGGTTCCCTAAGGGTTGGACTGACCTTGGGCCCTGCAGAGAACCTACA
2DL2 - #1 right	TTCATGGGCCTCCCCCTCCCTGGATGTCTAGATTGGATCTTGCTGGCAC
2DL2 - #2 left	GGGTTCCCTAAGGGTTGGACATGTCCTATGATCCTAGAGCCTTAGCTGAGGAGCTTCCTGCTGATGATGGAGAT
2DL2 - #2 right	AAGCATGGACAGATGCAGAGAGAAGACGAAGCTTGGGTGTGAGGGAGGTCTAGATTGGATCTTGCTGGCAC
2DL3 - #1 left	GGGTTCCCTAAGGGTTGGACYRCACAGTTGRATCACTGCGTTTTCACACAGAGAAAAATCACTCRCCCTT
2DL3 - #1 middle	CTCAGAGGCCCAAGACACCCCCAACAGATATCATCGTGTACACGGAACTTCCAAATGCTGAGCCCT
2DL3 - #1 right	GATCCAAAGTTGTCTCCTGCCCATGAGCACCACAGTCAGGCCTTGTCTAGATTGGATCTTGCTGGCAC
2DL3 - #2 left	GGGTTCCCTAAGGGTTGGACTGCTGCCTTGGGCCAGGGACCATCCTGTCTGTGAGGAACACACACCTGAGTGCTC
2DL3 - #2 middle	CCATCCTGCTTCCCCACATGGCCCTGAGCTCTCTGGCCTCTGCTTCGTGAGACTTACTTTTTTTGTTGC
2DL3 - #2 right	AGCACCAGCGATGAAGGAGAAAGAAGAGGAGGAGGATGAAGAGGATGTCTAGATTGGATCTTGCTGGCAC
2DL4 - #1 left	GGGTTCCCTAAGGGTTGGACCTGCTTCAGAACATGGCTCTCTGCTGGGGAGACACCCAA
2DL4 - #1 middle	TCTGCAGGCCCATAGTGTAACCCTGGTGCTCCTTCCCTTCCAGGACTCACCAAG
2DL4 - #1 right	ACATGCCAGGATGATGACCGTGGGTGACATGGAGTCTAGATTGGATCTTGCTGGCAC
2DL4 - #2 left	GGGTTCCCTAAGGGTTGGACCCGCTTAGAAAGAAGAAATGGGGAGAATCTTCTGAGCACAGGGAGGGAGGGGC
2DL4 - #2 middle	AGCTCAACATACTCCTCTCTGAGGCGGCATCTCCTTCTCCCCAAGGTGGTCAGGACAAGCCCTTCTGC
2DL4 - #2 right	TCTGCCTGGCCCAGCGCTGTGGTGCCTCAAGGAGGACACGTGACTCTTCGGTGGTCTAGATTGGATCTTGCTGGCAC
2DL5 - #1 left	GGGTTCCCTAAGGGTTGGACTCAGGTGTGAGGGGAGCTGTGACAAGGAAGAACCTCC
2DL5 - #1 middle	CTGAGGAAACTGCCTCTTCTTCCAGGTCTATT
2DL5 - #1 right	TGGGAAACCTTCACTCTCAGCCCAGCCGGGTCTAGATTGGATCTTGCTGGCGC
2DL5 - #2 left	GGGTTCCCTAAGGGTTGGACCGACCTACACATGCTTYRGCTCTCTCCATGACTC
2DL5 - #2 right	ACCCTATGAGTGGTCAGACCCGAGTGACCCGTCTAGATTGGATCTTGCTGGCAC
2DL5b left	GGGTTCCCTAAGGGTTGGACATGTTAGCACAGATTTTAGGCATCTCGTGTTCGGATAAAAATACATGAAAAGTCTTTCAC
2DL5b middle	GTTAGCACAGATTTTAGGCATCTTGTGTTCGGGAGGTTGGATCTGAGACGTGTTGTGAGTTGGTCATAGTGAAGGACGT
2DL5b right	GAGGTGCCAATTCTAGTGAGAACAATTTCCAGGAAGCCGTGTTCCGGTCTAGATTGGATCTTGCTGGCAC
2DP1 - #1 left	GGGTTCCCTAAGGGTTGGACCCAAGGTGGTCAGGACAAGCCCTTGCTGTCTGCCTGGCCCAGCTC
2DP1 - #1 right	TGTGGTGCCTCCAGGACATGTGATTCTTCGGTGTCTAGATTGGATCTTGCTGGCGC
2DP1 - #2 left	GGGTTCCCTAAGGGTTGGACCAGGGACCTACAGATGCTACGGTTCTGTTACTCACTCCCC
2DP1 - #2 right	CATCAGTTGTCAGCTCCCAGTGACCCTCTGGACATCGTCATGTCTAGATTGGATCTTGCTGGCAC
2DS1 - #1 left	GGGTTCCCTAAGGGTTGGACACAGGGCCCATGAAAAGGCTGTTCCAGAATATTATGTTGTAGAGCTCAGGGACAGGCA
2DS1 - #1 middle	CCCCATCTTCCTTTTACAGACTGAAGTTGTTAAACCCAAGATAAGAATGACACTGAAGAATCACATA
2DS1 - #1 right	TCCTGGAGGCACCACAGGGCTTGGCCAGTCTAGATTGGATCTTGCTGGCAC
2DS1 - #2 left	GGGTTCCCTAAGGGTTGGACTAGGAGACCGTGGAAAAGGCAATTCCCGA
2DS1 - #2 middle	CCCACTGGTGAAATGTGGTGCTGATTTT
2DS1 - #2 right	GACACTAAGTGGATGAAGCAGATGGATATAAGCGTCTAGATTGGATCTTGCTGGCAC
2DS2 left	GGGTTCCCTAAGGGTTGGACCGGCCGAGCACCCCAGGGTCCTCTCTTCCC
2DS2 right	AGTTTATGAGAGACTCCCTGACAGGACGTCTAGATTGGATCTTGCTGGCAC
2DS3 - #1 left	GGGTTCCCTACGGGTTGGACCATCACGATGTCCAGAGGGTCACTGGGAGCTGAA
2DS3 - #1 right	AACTGATAGGGGGAGTGAGGAACAGAGACCGTCTAGATTGGATCTTGCTGGCAC
2DS3 - #2 left	GGGTTCCCTAAGGGTTGGACCGAAAGAGCCGAAGCATCTGTAGGTTCCTCCT
2DS3 - #2 right	TGGGTGGCAGGGCCCAGAGGAAAGTCGGCCTGGAATGTCTAGATTGGATCTTGCTGGCAC

Table S1 continued
Overview probe sequences of the KIR MLPA technique.

Probe	Sequence (5'-3')
2DS4 - All left	GGGTTCCCTAAGGGTTGGACTCCCCTCTCTGTGCAGAAGGAAGTGCTCAAACATGACATCC
2DS4 - All right	GACCAACATTGCAGGATGACTGTCTCTTCTGATTTCACCAGGTGACCTGGGAGTCTAGATTGGATCTTGCTGGCAC
2DS4 - WT/trunc left	GGGTTCCCTAAGGGTTGGACCTTGGGCCCAGAGGAAAGTCRGCCTGGAATGTTCCGTKGAT
2DS4 - trunc middle	GCTGCGCACTGCAGGGAGCCTACGTTCATGGGCCTCCCCYTCCCTGGATAG
2DS4 - trunc right	ATGGAGCTGCAGGACAAGGTCACAGTCTAGATTGGATCTTGCTGGCAC
2DS4 - WT middle	GCTGCGCACTGCAGGGAGCCTACGTTCATGGGCCTCCCCYTCCCTGGATAGATGGTAC
2DS4 - WT right	CATGTCATAGGAGCTCCGGGAGCTGCAGGACAAGGWCACATTCTCTCTCTAGATTGGATCTTGCTGGCAC
2DS5 left	GGGTTCCCTACGGGTTGGACCGAGTAAACCGGAAAATTTTCATCTGCACAGAGAGGGGACGTTTAAC
2DS5 middle	CACACTTTGCCCCTCATTGGAGAGCACATTGATGGGGTCTCCAAGGG
2DS5 right	CAACTTCTCCCTCGGTCGCATGACACAAGACCTGGCATAGCGAATACGTCTAGATTGGATCTTGCTGGCAC
3DL1 - #1 left	GGGTTCCCTAAGGGTTGGACCCCTCAHGCCTCGYTGGACA
3DL1 - #1 middle	GATCCATGATGGGGTCTCCAAGGCCAATTTCTCCATCGGTCCCATGATGCT
3DL1 - #2 left	GGGTTCCCTAAGGGTTGGACTCAGCTCAGGTATGAGGGGAGCTATGACAAGGAAGAACCT
3DL1 - #2 middle	CCCTGAGGAAACTGCCTCTTCTCCTTCCAGGTCC
3DL1 - #2 right	ATATGAGAAACCTTCTCTCTCAGCCCAGCCGGGTCTAGATTGGATCTTGCTGGCAC
3DL1/S1 right	TGCCCTTGCAGGGACCTACAGATGCTACGGTTCTGGTCTAGATTGGATCTTGCTGGCAC
3DL2 - #1 left	GGGTTCCCTAAGGGTTGGAATCCACCCTAAGGTTTGGGGAKGGACTCACCCATGA
3DL2 - #1 right	GTGGCCAGGCCCCCTGCAGCAAGAAGAACCCTGTCTAGATTGGATCTTGCTGGCGC
3DL2 - #2 left	GGGTTCCCTAAGGGTTGGACCATGAAGCTCCTCAGCTATGGCTCTAGGATCATAAGACATGGGACAGACA
3DL2 - #2 middle	CGGGTTTTCCTCACCTGTGACAGAAACAAGCAGTGGGTCACTTGAGTTTGACCACACGCA
3DL2 - #2 right	GGGCAGGGCACGGAAAGAGCCGAAGCATCTGTAGTTCCCTCGTCTAGATTGGATCTTGCTGGCAC
3DL3 - #1 left	GGGTTCCCTACGGGTTGGATAGATGCTTCGGCTCTTTCCGTGCCCTGCCC
3DL3 - #1 right	CAYGCGTGGTCAGACCCGAGTGACCCGTCTAGATTGGATCTTGCTGGCAC
3DL3 - #2 left	GGGTTCCCTAAGGGTTGGACAAGGGTGAGAGGCAGGTCTGTATTCTCTCACCTA
3DL3 - #2 middle	CGACCACGATGTCCAGAGGGTCACTGGGAGCYGACAACTCATAGGGTA
3DL3 - #2 right	AGTGAGTGACAGAACCAAAGCATCTGTAGTCTAGATTGGATCTTGCTGGCAC
3DP1 - #1 left	GGGTTCCCTAAGGGTTGGACACCATGATCACCAGGGGGTTGCTGGGTGCTGACCACCCAGTGAGGA
3DP1 - #1 right	AGTGTGGGTGTGAACCCCGACATCTGTAGGTCCCTGTCTAGATTGGATCTTGCTGGCGC
3DP1 - #2 left	GGGTTCCCTAAGGGTTGGACGCTGAGGCCTGGAAAGGAATAGAGGGAGGGAGTGCCACATC
3DP1 - #2 right	CTCCTCTCTAAGGTGGCGCCTCCTTCTCCCCCAGGTGTCTAGATTGGATCTTGCTGGCAC
3DS1 - #1 left	GGGTTCCCTAAGGGTTGGACTTGTTCATCAGAATCCTGGAGAGAGGGAAATGCTGAGTGAGGGAGGGTGCTCACATTTTTC
3DS1 - #1 middle	AGGACTCTTTGGGAATAACACTAGCCACGAGGCTGGGCCGAGGAGCACCTACCTCGCTGTTCAC
3DS1 - #1 right	TTCTGTTCCCTGCAGGCTCTTGGTCCATTACAGCAGCATCTGTAGGAGACGTCTAGATTGGATCTTGCTGGCGC
3DS1 - #2 left	GGGTTCCCTAAGGGTTGGACACTTCTTTCTGCACAAAGAGTGGATCTCTAAGG
3DS1 - #2 middle	ACCCCTCACGCCTCGTTGGACAGATCCATGATGGGGTCTCCAAGGCCAATTTCTCCATCGGTTCCATGATGCG
