# Supplementary material for: Extensive Variation in Gene Copy Number at the Killer Immunoglobulin-Like Receptor Locus in Humans
Source: PLoS One. 2013 Jun 28;8(6):e67619. doi: 10.1371/journal.pone.0067619 (PMC3695908; doi:10.1371/journal.pone.0067619)
Supplement: Table S2 — Overview control probe sequences of the KIR MLPA technique. (PDF) [file pone.0067619.s006.pdf]

Table S2
Overview control probe sequences of the KIR MLPA technique.

Probe	Sequence (5'-3')
Control 1 - left	GGGTTCCCTAAGGGTTGGACCAGCCAGCAGCAGCCCCAAGCTGATAAGATTAATCTAAAGAGCAAATTATGGT
Control 1 - middle	GTAATTTCCTATGCTGAAACTTTGTAGTTAATTTTTTAAAAAGGTTTCATTTTCCTATTGGTCTGATTT
Control 1 - right	CACAGGAACATTTTACCTGTTTGTGAGGCATTTTTTCTCCTGGTCTAGATTGGATCTTGCTGGCAC
Control 2 - left	GGGTTCCCTAAGGGTTGGATAGAGGGCTCCAGGTTATCCGGGCTC
Control 2 - right	TGCTTCTGCCGCCGCCGTCGGGGGTCTAGATTGGATCTTGCTGGCAC
Control 3 - left	GGGTTCCCTAAGGGTTGGATTCAGGAGTGATACTTCACAGATCCTGGAGGAA
Control 3 - right	AACATCCCAGTCCTTAAGGCCAAACTGAGTCTAGATTGGATCTTGCTGGCAC
Control 4 - left	GGGTTCCCTAAGGGTTGGACTGCGTGAGACCAAGCGCCGTCATGAGACCCGACTGGTG
Control 4 - right	GAGATTGACAATGGGAAGCAGCGTGAGTTTGAGAGTCTAGATTGGATCTTGCTGGCAC
Control 8 - left	GGGTTCCCTAAGGGTTGGACCTGCTCCCAGTAGGGTCAGCATCTGGACCCCAGGCTGA
Control 8 - middle	GAGTCAGGCTCTGATTCCAGATCTAGCCTCCATCATGAAGAAGCTCTTGACCAAGTATG
Control 8 - right	ACAACCTCTTTGAGACGTCCTTTCCCTACTCCATGTCTAGATTGGATCTTGCTGGCAC
Control 9 - left	GGGTTCCCTAAGGGTTGGACGCAGGACAGAAGGAGCAAGCTGTGGAATGGTATAAGAAAG
Control 9 - middle	GTATTGAAGAACTGGAAGAAGGAATAGCTGTTATAGTTACAGGACAAGGTAAGATTGTAT
Control 9 - right	TTGTTTATAGCCATCCCAAATTATGATATATTCACACTCTAGATTGGATCTTGCTGGCAC
Control 10 - left	GGGTTCCCTAAGGGTTGGACCTTCCCCATTGGTTTGTTATTGCAGATGAAGTGGAAAGGGAAGGACCTCTTTGATTTG
Control 10 - middle	GTGTGCCGGACTCTGGGGCTCCGAGAAACCTGGTTCTTTGGACTGCAGTACACAATCAAGGACACAGTG
Control 10 - right	GCCTGGCTCAAAATGGACAAGAAGGTTGGGCTAGAACTCGATGAAACTGGTGCTCTAGATTGGATCTTGCTGGCAC
