# Supplementary material for: Extensive Variation in Gene Copy Number at the Killer Immunoglobulin-Like Receptor Locus in Humans
Source: PLoS One. 2013 Jun 28;8(6):e67619. doi: 10.1371/journal.pone.0067619 (PMC3695908; doi:10.1371/journal.pone.0067619)
Supplement: Table S3 — KIR MLPA technique probe distribution over 3 mixes. (PDF) [file pone.0067619.s007.pdf]

Table S3
KIR MLPA technique probe distribution over 3 mixes.

Mix 1		Mix 2		Mix 3		KIR controls		
Probe	Product Length	Probe	Product Length	Probe	Product Length	Probe	Target	Product Length
2DL1 - #1 left	190	2DL1 - #2 left	97	2DL1 - #2 left	97	Control 1 - left	IL-4	208
2DL1 - #1 middle		2DL1 - #2 right		2DL1 - #2 right		Control 1 - middle		
2DL1 - #1 right		2DL2 - #2 left	145	2DL3 - #2 left	214	Control 1 - right		
2DL2 - #1 left	96	2DL2 - #2 right		2DL3 - #2 middle		Control 2 - left	FGF3	92
2DL2 - #1 right		2DL3 - #1 left	204	2DL3 - #2 right		Control 2 - right		
2DL3 - #1 left	204	2DL3 - #1 middle		2DL4 - #1 left	170	Control 3 - left	BCAS4	104
2DL3 - #1 middle		2DL3 - #1 right		2DL4 - #1 middle		Control 3 - right		
2DL3 - #1 right		2DL3 - #2 left	214	2DL4 - #1 right		Control 4 - left	LMNA	116
2DL4 - #1 left	170	2DL3 - #2 middle		2DL5b left	229	Control 4 - right		
2DL4 - #1 middle		2DL3 - #2 right		2DL5b middle		Control 8 - left	GALT	175
2DL4 - #1 right		2DL4 - #2 left	218	2DL5b right		Control 8 - middle		
2DL5 - #1 left	142	2DL4 - #2 middle		2DP1 - #1 left	121	Control 8 - right		
2DL5 - #1 middle		2DL4 - #2 right		2DP1 - #1 right		Control 9 - left	SPG4	180
2DL5 - #1 right		2DL5 - #2 left	108	2DS1 - #2 left	134	Control 9 - middle		
2DP1 - #1 left	121	2DL5 - #2 right		2DS1 - #2 middle		Control 9 - right		
2DP1 - #1 right		2DL5b left	229	2DS1 - #2 right		Control 10 - left	NF2	223
2DS1 - #1 left	195	2DL5b middle		2DS3 - #1 left	108	Control 10 - middle		
2DS1 - #1 middle		2DL5b right		2DS3 - #1 right		Control 10 - right		
2DS1 - #1 right		2DP1 - #2 left	125	2DS5 left	185			
2DS3 - #1 left	108	2DP1 - #2 right		2DS5 middle				
2DS3 - #1 right		2DS1 - #2 left	134	2DS5 right				
2DS4 - All left	137	2DS1 - #2 middle		3DL1 - #1 left	150			
2DS4 - All right		2DS1 - #2 right		3DL1 - #1 middle				
2DS4 - WT/trunc left	160	2DS2 left	101	3DL1/S1 right				
2DS4 - trunc middle		2DS2 right		3DL3 - #1 left	100			
2DS4 - trunc right		2DS3 - #2 left	112	3DL3 - #1 right				
2DS5 left	185	2DS3 - #2 right		3DP1 - #2 left	121			
2DS5 middle		2DS4 - WT/trunc left	190	3DP1 - #2 right				
2DS5 right		2DS4 - WT middle						
3DL1 - #1 left	150	2DS4 - WT right						
3DL1 - #1 middle		3DL1 - #2 left	150					
3DL1/S1 right		3DL1 - #2 middle						
3DL2 - #1 left	111	3DL1 - #2 right						
3DL2 - #1 right		3DL2 - #2 left	195					
3DL3 - #1 left	100	3DL2 - #2 middle						
3DL3 - #1 right		3DL2 - #2 right						
3DP1 - #1 left	125	3DL3 - #2 left	154					
3DP1 - #1 right		3DL3 - #2 middle						
3DS1 - #1 left	219	3DL3 - #2 right						
3DS1 - #1 middle		3DP1 - #2 left	121					
3DS1 - #1 right		3DP1 - #2 right						
		3DS1 - #2 left	185					
		3DS1 - #2 middle						
		3DL1/S1 right						
