# Supplementary material for: Extensive Variation in Gene Copy Number at the Killer Immunoglobulin-Like Receptor Locus in Humans
Source: PLoS One. 2013 Jun 28;8(6):e67619. doi: 10.1371/journal.pone.0067619 (PMC3695908; doi:10.1371/journal.pone.0067619)
Supplement: Table S4 — Copy number determination by KIR MLPA technique. (PDF) [file pone.0067619.s008.pdf]

Table S4
Copy number determination by KIR MLPA technique.

Gene detected	Determining CNV
2DL1	Average of all probes
2DL2	Average of all probes
2DL3	Average of #2 in Mix 2 and 3
2DL4	Average of all probes
2DL5 *	2DL5 #2 in Mix 2
2DS1	Average of #2 multiplied by 2 in Mix 2 and 3
2DS2	2DS2 in Mix 2
2DS3	Average 2DS3 #2 in Mix 2 and 2DS3 #1 in Mix 3
2DS4 All	2DS4 All and independently copies of WT + trunc
2DS4 WT	2DS4 WT
2DS4 truncated	2DS4 trunc multiplied by 2
2DS5	Average of all probes
2DP1	Average of all probes
3DL1	Average of 3DL1 #1 in Mix 1 and 3
3DL2	Average of all probes
3DL3	Average of all probes
3DS1	Average of all probes
3DP1	3DP1 #1 in Mix1

* Detects both 2DL5A and B
