# Supplementary material for: Extensive Variation in Gene Copy Number at the Killer Immunoglobulin-Like Receptor Locus in Humans
Source: PLoS One. 2013 Jun 28;8(6):e67619. doi: 10.1371/journal.pone.0067619 (PMC3695908; doi:10.1371/journal.pone.0067619)
Supplement: Table S5 — A sequence alignment of the short tandem repeat region in intron 4 of most KIRs. (PDF) [file pone.0067619.s009.pdf]

**Table S5**

**A sequence alignment of the short tandem repeat region in intron 4 of most KIRs.**

```
3DS1_450      -CCAAATAGAACTAGAGAGACTGAGAGGCAGAGAAAGACAAGGAGACGGAGAGAGAGAGATGATAGATGGATAGATA-----------------GACGTAGATAGATGATAAATA---GG
3DL1_422      -CCAAATAGAACTAGAGAGACTGAGAGGCAGAGAAAGACAAGGAGACGGAGAGAGAGAGATGATAGATGGATAGATA-----------------GACGTAGATAGATGATAAATA---GG
3DL2_447      -CCAAATAGAACTAGAGAGACTGAGAGGCAGAGAAAGACAAGGAGATGGAGAGAGACAGATGATAGATGGATAGAYA-----------------GATATAGATAGATGATAAATA---GG
2DL1_405      -CCAAAGAGAACTAGAGAGACCGAGAGGCAGAGCAAGACA------------------GATGATAGATGGATAGATA---------------------TAGATAGATGATAAATA---GG
2DS1_405      -CCAAAGAGAACTAGAGAGACCGAGAGGCAGAGCAAGACA------------------GATGATAGATGGATAGATA---------------------TAGATAGATGATAAATA---GG
2DS5_401      -CCAAAGAGAACTAGAGAGACCGAGAGGCAGAGCAAGACA------------------GATGATAGATGGATAGATA---------------------TAGATAGATGATAAATA---GG
2DS4_424      -CCAAAGAGAACTAGAGAGACCGAGAGGCAGAGCAAGACA------------------GATGATAGATGGATAGATACAGATAGATGATGGATAGATATAGATAGATGATATATA---GG
3DP1_401      -CCAAAGAGAACTAGAGAGACCGAGAGGCAGAGCAAGACA------------------GATGATAGATGGATAGATA---------------------TAGATAGATGATAAATA---GG
2DL2_391      -CCAAAGAGAGCTAGAGAGACCGAGAGGCAGAGCAATACA------------------GATGATAGATGGATAGATA---------------------TAGATAGATGATAAATA---GG
2DL3_391      -CCAAAGAGARCTAGAGAGACCGAGAGGCAGAGCAAGACA------------------GATGATAGATGGATAGATA---------------------TAGATAGATGATAAATA---GG
2DS2_395      -CCAAAGAGAACTAGAGAGACCGAGAGGCAGAGCAAGACA------------------GATGATAGATGGATAGATA---------------------TAGATAGATGATAAATA---GG
2DS3_392      -CCAAAGAGAACTAGAGAGACCGAGAGGCAGAGCAAGACA------------------GATGATAGATGGATAGATA---------------------TAGATAGATGATAAATA---GG
2DP1_395      -CCAAAGAGAACTAGAGAGACCGAGAGGCAGAGCAAGACA------------------GATGATAGAWGGWTAGATA---------------------TAGATAGATGATAAATA---GG
3DL3_416      CCAAAAGGGAACTAGAGAGACTGAGAGGCAGAGAAAGACAAGGAGATGGAGAGAGACAGATGATAGATGGATAGATA-----------------GATATAGATAGATGAAAGATAAAAGG
               * ***  ** ********** *********** ** ***                  ********* ** **** *                     *********** * ***   **

3DS1_450      TAGATGATAGATAATGGA----TTGGTTATAGATACATAGATGATGACTGAT----------------AGATGATACATAGAGATGATGATGATGACGATGATGATGATAGACACATAGA
3DL1_422      TAGATGATAGATAATGGA----TTGGTTATAGATACATAGATGATGACTGAT----------------AGATGATACATAGAGATGA---------CGATGATGATGATAGACACATAGA
3DL2_447      TAGATGATAGATAATGGA----TAGGTTATAGATACATAGATGATGATTGAT----------------AGATGATACATAGAGATGATGATGATGATGATGATGAAGATAGATAGATAGA
2DL1_405      TAGATGATAGATAATAG--------GTTATAGATACATAGATGATGATTGATTGATTCATT----AATAGATGAGACATAGAGATGA---------TGATGATGAAGACASATAGATAGA
2DS1_405      TAGATGATAGATAATAG--------GTTATAGATACATAGATGATGATTGATTGATTCATT----AATAGATGAGACATAGAGATGA---------TGATGATGAAGACAGATAGATAGA
2DS5_401      TAGATGATAGATAATAG--------GTTATAGATACATAGATGATGATTGATTGATTCATT----AATAGATGAGACATAGAGATGA---------TGATGATGAAGACAGATAGA----
2DS4_424      TAGATGATAGATAATAG--------GTTATAGATACATAGATGATGATTGATTGATTCATT----AATAGATGATACATAGAGATGA---------TGATGATGAAGATAGATGGATAGA
3DP1_401      TAGATGATAGATAATAG--------GTTATAGATACATAGATGATGATCGATTCATTCATTGATTAATCGATGATACATAGAGATGA---------TGAAGATGAAGATA----GATAGA
2DL2_391      TAGATGATAGATAATAG--------GTTAAAGATACATAGATGATGATTGATTGATTCATT----AATAGATAATACATAGAGATGA---------TGATGATGAAGACA----GATA--
2DL3_391      TAGATGATAGATAATAG--------GTTAAAGATACATAGATGATGATTGATTGATTCATT----AATAGATAATACATAGAGATGA---------TGATGATGAAGACA----GATA--
2DS2_395      TAGATGATAGATAATAG--------GTTAAAGATACATAGATGATGATTGATTGATTCATT----AATAGATAATACATAGAGATGA---------TGATGATGAAGACA----GATAGA
2DS3_392      TAGATGATAGATACTAG--------GTTATAGATACATAGATGATGATTGATTGATTCATT----AATAGATGAGACGTAGAGATGA---------TGATGA---AGACA----GATAGA
2DP1_395      TAGATGATAGATAATAG--------GTTAAAGATACATAGATGATGATTGATTGATTCATT----AATAGATGAGACATAGAGATGA---------TGATGATGAAGACA----GATAGA
3DL3_416      TAGATGATAGATAATAGAGAGACAGGTGATAGACAAATAGATGATGAATGACTGAT------------AGATGATATAGATAGACAA--------GTAGAAAGACAGACAGAT-GATATA
              ************* * *        ** * *** * ***********  **                  *** * *   * ***  *              *    ** *     *

3DS1_450      ---TATATACATAGAT-----GATACATAAAT-------AGAGACAGAGAGGCAGACA----GAGAGGTAATAGAGAGAGAGATAGATG-ATACATA--TATAGATAATAGATGATTGAT
3DL1_422      ---TATATACATAGAT-----GATACATAAAT-------AGAGACAGAGAGGCAGACA----GAGAGGTAATAGAGAGAGAGATAGATG-ATACATA--TATAGATAATAGATGATTGAT
3DL2_447      AGACACATATATAAATATATAGATACATAGATGATACATAGAGACTGACAGGCAGACA----GAGAGGTAATAGAGAGAGAGAGAGATG-ATACATAGATACAGATAATACATAGATGAT
2DL1_405      ---------------T-----AATACATA-----------GAGATACAGAGGCAGACATAGAG--AAATCATAGAGAGAGAGA--GATG-ATACATAGATATAGATAATAGATGATTGAT
2DS1_405      ---------------T-----AATACATA-----------GAGATACAGAGGCAGACATAGAG--AAATCATAGAGAGAGAGA--GATG-ATACATAGATATAGATAATAGATGATTGAT
2DS5_401      ---------------T-----AATACATA-----------GAGATAGAGAGGCAGACATAGAG--AAATCATAGAGAGAGAGA--GATG-ATACACAGATATAGATAATAGATGATTGAT
2DS4_424      ---------------T-----AATACATA-----------GAGATAGAGAGGAAGACAAAGAGAGAAATAATAGAGAGAGAGA--GATG-ATACATATATATAGATAATAGATGATTGAC
3DP1_401      ---------------T-----AATACATA-----------GAGATAGAGAGGCAGACAAAGAG--AAATCATAGAGAGAGAGA--GATG-ATACATAGATATAGATAATAGATGATTTTT
2DL2_391      ----------------------ATACGTA-----------CAGATAGAGAGGCAGACA----G--AAATCATAGAGAGAGAG----ATG-ATACATACATATAAATAACAGATGATTGAT
2DL3_391      ----------------------ATACGTA-----------CAGATAGAGAGGCAGACA----G--AAATCATAGAGAGAGAG----ATG-ATACATACATATAAATAACAGATGATTGAT
2DS2_395      ---------------T-----AATACGTA-----------CAGATAGAGAGGCAGACA----G--AAATCATAGAGAGAGAG----ATG-ATACATACATATAAATAACAGATGATTGAT
2DS3_392      ---------------T-----AATACATA-----------GAGATAGAGAGGCAGACA----G--AAGTCATAGAGAGAGAG----ATG-ATACATAGATATAGATAACAGATGATTGAT
2DP1_395      ---------------T-----AATACATA-----------GAGATAGAGAGGCAGACA----G--AAGTCATAGAGAGAGAG----ATG-ATACATAGATATAGATAACAGATGATTGAT
3DL3_416      ---------------TA----AATAGATATAG-------AGAGATAGAAAGACAGATAAAC-ACATGATGATAGATGGATAG----ATGCATACATACATACAT-TGATTGATAGATGAT
                                    ***  **            ***   * **  *** *          * *****  ** **    *** ***** *  ** *  * *   **   *

3DS1_450      GGATAGATAGACAGATAGACAATTGATAGAGAGATAGATAAGTGATACATAAATATAGATGATAGATA----ATTTGTAGATAGACACAAAATAGATA-------------------AAT
3DL1_422      GGATAGATAGACAGAYAGACAATTGATAGAGAGATAGATAAGTGATACATAAATATAGATGATAGATA----ATTTGTAGATAGACACAAAATAGATA-------------------AAT
3DL2_447      TGATGGATAGACAGATAGACAATTGATAGA--------TAAATGATACATAGATATAGATGACAGATA----ATTTGTAGATAGACACAAAATAGATAGATAGA------------TAAT
2DL1_405      GGAT--------AGATAGACAATTGATGGA----TAAATAGATGATATATAGATATAGATGACAGGTAGAGAATTTGTAGATAGGCACCGAATAGATAAATAGATAGATCGATAGATAAT
2DS1_405      GGAT--------AGATAGACAATTGATGGA----TAAATAGATGATATATAGATATAGATGACAGGTAGAGAATTTGTAGATAGGCACCGAATAGATAAATAGATAGATCGATAGATAAT
2DS5_401      GGAT--------AGATAGAAAATTGATAGA----TAAATAGATGATATATAGATATAGATGACAGGTAGAGAATTTGTAGATAGGCACGGAATAGATAAATAGATAGATCGATAGATAAT
2DS4_424      GGAT--------AG----ACAATTGATAGA----TAAATAGATGATATATAGATATAGATGACAGGTAGAGAATTTGTAGATAGGCACCGAATAGATAAATAGATGGATTGATAGATAAT
3DP1_401      GGAT--------AG----ACAATTGATAGA----TAAATAGATTATATATAGATATAGATGACAGGTAGAGAATTTGTAGATAGGCACCAGATAGATAAATAGATATATCGATAGATAAT
2DL2_391      GGAT--------AGATAGACAACTGATAGA----TACATAGATGATATATAGATATAGATGACAGGTAGAGAATTTGTAGATAGGCACCGAATAGATAAATAGATAGATCGACAGATAAT
2DL3_391      GGAT--------AGATAGACAAGTGATAGA----TACATAGATGATATATAGATATAGATGACAGGTAGAGAATTTGTAGATAGRCACCGAATAGATAAATAGATAGATCGACAGATAAT
2DS2_395      GGAT--------AGATAGACAACTGATAGA----TACATAGATGATATATAGATATAGATGACAGGTAGAGAATTTGTAGATAGGCACCGAATAGATAAATAGATAGATCGACAGATAAT
2DS3_392      GGAT--------AGATAGACAAGTGATAGA----TACATAGATGATATATAGATATAGATGACAAGTAGAGAATTTGTAGATAGGCACCGAATAGATAAATAGATAGATCAACAGATAAT
2DP1_395      GGAT--------AGATAGACAAGTGATAGA----TACATAGATGATATATAGAYATAGATGACAGGTAGAGAATTTGTAGATAGGCACCGAATAGATAAATAGATAGATCGAYAGATAAT
3DL3_416      AGATA-----ACAGAGAGATAGGTCATAGA----TACACAGATGATG-------ATAGATGATAGATAC---ATACATAGATAAATGATAGATCGATCAATAGA------------TAGT
               ***        **    * *  * ** **         *  * **        ******** *  **    **   ******        ** ***                    * *

3DS1_450      AGATAGATCGATAGATAATAGATAGAAATGTGCAGAAAGTTATGAACAAGACAGAA 450
3DL1_422      AGATAGA-------------------AATGTGCAGAAAGTTATGAACAAGACAGAA 422
3DL2_447      AGATAGA-------------------AATATGCAGAAAGTTATGAACAAGACAGAA 447
2DL1_405      AGATAGA-------------------AATATGCAGAAAGTTATGRACAGGACACAA 405
2DS1_405      AGATAGA-------------------AATATGCAGAAAGTTATGAACAGGACACAA 405
2DS5_401      AGATAGA-------------------AATATGCAGAAAGTTATGAACAGGACACAA 401
2DS4_424      AGATAGA-------------------AATATGCAGAAAGTTATGAACGGGACACAA 424
3DP1_401      AGATAGA-------------------AATATGCAGAAAGTTATGAACAGGACACAA 401
2DL2_391      AGATAGA-------------------AATATGCAGAAAGTTATGAACAGGACACAA 391
2DL3_391      AGATAGA-------------------AATATGCAGAAAGTTATGAACAGGACACAA 391
2DS2_395      AGATAGA-------------------AATATGCAGAAAGTTATGAACAGGACACAA 395
2DS3_392      AGATAGA-------------------AATATGCAGAAAGTTATGAACAGGACACAA 392
2DP1_395      AGATAGA-------------------AATATGCAGAAAGTTATGAACAGGACACAA 395
3DL3_416      AGATAGA-------------------AATATGCAGAAAGTTATGAGCAAGACAGAA 416
              *******                   *** **************  *  **** **
```
