# Supplementary material for: Extensive Variation in Gene Copy Number at the Killer Immunoglobulin-Like Receptor Locus in Humans
Source: PLoS One. 2013 Jun 28;8(6):e67619. doi: 10.1371/journal.pone.0067619 (PMC3695908; doi:10.1371/journal.pone.0067619)
Supplement: Table S6 — KIR gene copy number distribution in a cohort of healthy individuals. (PDF) [file pone.0067619.s010.pdf]

Table S6
KIR gene copy number distribution in a cohort of healthy individuals.

KIR Gene	Copies				
	**0**	**1**	**2**	**3**	**>3**
**2DL1**	4.2%	21.7%	62.5%	11.7%	0.0%
**2DL2**	46.7%	45.8%	7.5%	0.0%	0.0%
**2DL3**	12.5%	51.7%	35.0%	0.8%	0.0%
**2DL4**	0.0%	3.3%	88.3%	8.3%	0.0%
**2DL5**	43.7%	39.5%	16.0%	0.8%	0.0%
**2DS1**	62.5%	31.7%	5.8%	0.0%	0.0%
**2DS2**	46.2%	49.6%	4.2%	0.0%	0.0%
**2DS3**	68.3%	26.7%	5.0%	0.0%	0.0%
**2DS4all**	3.4%	26.9%	68.1%	1.7%	0.0%
**2DS4wt**	60.8%	32.5%	6.7%	0.0%	0.0%
**2DS4trunc**	18.3%	50.0%	30.8%	0.8%	0.0%
**2DS5**	66.7%	32.5%	0.8%	0.0%	0.0%
**2DP1**	4.2%	24.2%	58.3%	13.3%	0.0%
**3DL1**	3.3%	34.2%	59.2%	3.3%	0.0%
**3DL2**	0.0%	0.0%	99.2%	0.8%	0.0%
**3DL3**	0.0%	0.0%	99.2%	0.8%	0.0%
**3DS1**	58.3%	17.5%	20.0%	4.2%	0.0%
**3DP1**	0.0%	15.0%	84.2%	0.8%	0.0%

n=120
